# Concepts, Control, and Context: A Connectionist Account of Normal and Disordered Semantic Cognition

**DOI:** 10.1037/rev0000094

**Published:** 2018-04

**Authors:** Paul Hoffman, James L. McClelland, Matthew A. Lambon Ralph

**Affiliations:** 1 Neuroscience and Aphasia Research Unit, University of Manchester, and Centre for Cognitive Ageing and Cognitive Epidemiology, Department of Psychology, University of Edinburgh; 2 Department of Psychology, Center for Mind, Brain and Computation, Stanford University; 3 Neuroscience and Aphasia Research Unit, University of Manchester

**Keywords:** semantic diversity, imageability, parallel distributed processing, semantic dementia, semantic aphasia

## Abstract

Semantic cognition requires conceptual representations shaped by verbal and nonverbal experience and executive control processes that regulate activation of knowledge to meet current situational demands. A complete model must also account for the representation of concrete and abstract words, of taxonomic and associative relationships, and for the role of context in shaping meaning. We present the first major attempt to assimilate all of these elements within a unified, implemented computational framework. Our model combines a hub-and-spoke architecture with a buffer that allows its state to be influenced by prior context. This hybrid structure integrates the view, from cognitive neuroscience, that concepts are grounded in sensory-motor representation with the view, from computational linguistics, that knowledge is shaped by patterns of lexical co-occurrence. The model successfully codes knowledge for abstract and concrete words, associative and taxonomic relationships, and the multiple meanings of homonyms, within a single representational space. Knowledge of abstract words is acquired through (a) their patterns of co-occurrence with other words and (b) acquired embodiment, whereby they become indirectly associated with the perceptual features of co-occurring concrete words. The model accounts for executive influences on semantics by including a controlled retrieval mechanism that provides top-down input to amplify weak semantic relationships. The representational and control elements of the model can be damaged independently, and the consequences of such damage closely replicate effects seen in neuropsychological patients with loss of semantic representation versus control processes. Thus, the model provides a wide-ranging and neurally plausible account of normal and impaired semantic cognition.

Our interactions with the world are suffused with meaning. Each of us has acquired a vast collection of semantic knowledge—including the meanings of words and the properties of objects—which is constantly called upon as we interpret sensory inputs and plan speech and action. In addition to storing such conceptual information in a readily accessible form, we must call upon different aspects of knowledge to guide behavior under different circumstances. The knowledge that books are heavy, for example, is irrelevant to most of our interactions with them but becomes important when one is arranging a delivery to a library. These twin, intertwined abilities—the representation of acquired knowledge about the world and the controlled, task-oriented use of this knowledge—we refer to as *semantic cognition*.

The representation of semantic knowledge has long been the target of statistical and computational modeling approaches. One popular perspective, prevalent in cognitive neuroscience, holds that representations of object concepts arise from associations between their key verbal and nonverbal properties (Barsalou, 1999; Damasio, 1989; Martin, 2016; Patterson, Nestor, & Rogers, 2007; Pulvermuller, 2001; Simmons & Barsalou, 2003; Tyler & Moss, 2001). Another, rooted in computational linguistics, holds that semantic representation develops through sensitivity to the distributional properties of word usage in language (Andrews, Vigliocco, & Vinson, 2009; Firth, 1957; Griffiths, Steyvers, & Tenenbaum, 2007; Jones & Mewhort, 2007; Landauer & Dumais, 1997; Lund & Burgess, 1996; Rohde, Gonnerman, & Plaut, 2006). To date, these two approaches have made limited contact with one another. However, as we will demonstrate in the present work, these approaches are mutually compatible and considerable theoretical leverage can be gained by combining them. The second element of semantic cognition—its flexible and controlled use—has been investigated extensively in functional neuroimaging, transcranial magnetic stimulation and neuropsychological studies (Badre & Wagner, 2002; Gold et al., 2006; Jefferies, 2013; Jefferies & Lambon Ralph, 2006; Robinson, Shallice, Bozzali, & Cipolotti, 2010; Thompson-Schill, D’Esposito, Aguirre, & Farah, 1997) but has rarely been incorporated formally into computational models.

In this article, we present an implemented computational model that synthesizes the two distinct approaches to semantic representation and, furthermore, we propose a mechanism by which control processes interact with the knowledge store. Our primary tests of this model were its ability: (a) to generate a unified account of semantic representation and control spanning concrete and abstract items; and (b) to account for the contrastive impairments observed in two neuropsychological syndromes, semantic dementia and semantic aphasia, which have been attributed to representational and control damage, respectively (Jefferies & Lambon Ralph, 2006; Rogers, Patterson, Jefferies, & Lambon Ralph, 2015). The main strengths of our model are (a) its ability to represent a range of semantic information, including the meanings of abstract as well as concrete words, in a perceptually embodied and context-sensitive format, and (b) its ability to regulate activation of this knowledge in a way that meets changing task demands.

The article is structured as follows. We begin by considering the key challenges in knowledge representation that motivated this work. We describe the architecture of the model and illustrate how it meets these challenges. We then move on to consider the important but neglected issue of semantic control and describe how we have implemented a controlled retrieval process, which interacts with the knowledge store to direct semantic processing in a task-appropriate fashion. With these representational and control elements in place, we next present three simulations of performance on semantic tasks. We demonstrate that damage to the model’s representations and control processes induces divergent patterns of performance that closely replicate those of patients with hypothesized deficits in these abilities. We conclude by considering implications for theories of the neural basis of semantic cognition and by noting some challenges for future work.

## Part 1: Representation of Semantic Knowledge

In cognitive neuroscience, there is widespread agreement that verbal, sensory, and motor experience, and the brain regions that represent such information, play an integral role in conceptual representation (Allport, 1985; Barsalou, 2008; Binder & Desai, 2011; Kiefer & Pulvermuller, 2012; Martin, 2016; Paivio, 1986; Lambon Ralph, Jefferies, Patterson, & Rogers, 2017). This *embodied semantics* position is supported by functional neuroimaging studies indicating that particular sensory and motor processing regions are activated when people process concepts which are linked to them (Chao, Haxby, & Martin, 1999; Goldberg, Perfetti, & Schneider, 2006; Kellenbach, Brett, & Patterson, 2001; Martin, Haxby, Lalonde, Wiggs, & Ungerleider, 1995; Thompson-Schill, Aguirre, D’Esposito, & Farah, 1999) and by neuropsychological and neurostimulation studies that link impairments in sensory-motor (S-M) processing with deficits for particular classes of semantic knowledge (Campanella, D’Agostini, Skrap, & Shallice, 2010; Farah & McClelland, 1991; Pobric, Jefferies, & Lambon Ralph, 2010; Warrington & Shallice, 1984). For example, damage to frontoparietal regions involved in representing actions disproportionately affects the semantic representations of tools and other manipulable objects (Buxbaum & Saffran, 2002). The degree of embodiment varies across theories (Meteyard, Cuadrado, Bahrami, & Vigliocco, 2012), with the most strongly embodied approaches proposing little distinction between the processes involved in direct S-M experience and those involved in representing knowledge acquired from such experiences (e.g., Gallese & Lakoff, 2005). Other theories hold that activation of S-M information is necessary but not sufficient for semantic representation, and that an additional, transmodal layer of representation is also needed (Binder, 2016; Blouw, Solodkin, Thagard, & Eliasmith, 2015; Damasio, 1989; Mahon & Caramazza, 2008; Patterson et al., 2007; Simmons & Barsalou, 2003). This rerepresentation is thought to be necessary because the mapping between the observable properties of objects and their conceptual significance is complex and nonlinear. As such, the development of coherent, generalizable conceptual knowledge requires integration of information from multiple modalities through a shared transmodal hub (Lambon Ralph, Sage, Jones, & Mayberry, 2010).

Rogers et al. (2004) provided a demonstration of the importance of transmodal representation, in an implemented neural network model known as the hub-and-spoke model, which is the starting point for the present work. The model consisted of several sets of “spoke” units representing sensory and verbal elements of experience. There were also a set of hidden units (the hub) which did not receive external inputs but instead mediated between the various spokes. The model’s environment consisted of names, verbal descriptions, and visual properties for 48 different objects. When presented with a particular input (e.g., the name *dog*), it was trained to activate other forms of information associated with that concept (its visual characteristics and verbal description) by propagating activation through the hub. During training, a learning algorithm applied slow, incremental changes to the connections between units, such that over time the network came to activate the correct information for all of the stimuli. In so doing, it developed distributed patterns of activity over the hub units that represented each of the 48 concepts. The similarity structure among these representations captured the underlying, multimodal semantic structure present in the training set.

To test the model further, Rogers et al. (2004) progressively removed the hub unit connections, which resulted in increasingly impaired ability to activate the appropriate information for each concept. These impairments closely mimicked the deficits observed in patients with semantic dementia (SD). SD is a form of frontotemporal dementia in atrophy centered on the anterior temporal lobes accompanies a selective erosion of all semantic knowledge—verbal and nonverbal (Hodges & Patterson, 2007; Hodges, Patterson, Oxbury, & Funnell, 1992; Snowden, Goulding, & Neary, 1989). SD patients exhibit deficits across a wide range of tasks that require semantic knowledge, including naming pictures, understanding words, using objects correctly, and identifying objects from their tastes and smells (Bozeat, Lambon Ralph, Patterson, Garrard, & Hodges, 2000; Hodges, Bozeat, Lambon Ralph, Patterson, & Spatt, 2000; Luzzi et al., 2007; Piwnica-Worms, Omar, Hailstone, & Warren, 2010). Deficits in SD have long been considered to result from damage to a central store of semantic representations (Warrington, 1975). Damage to the hub component of the Rogers et al. (2004) model produced the same pattern of multimodal impairment.

The close correspondence between the deficits of SD patients and the performance of the damaged hub-and-spoke model suggest that damage to the transmodal “hub” is the root cause of these patients’ deficits. Indeed, the pervasive semantic deficits in SD have been linked with damage to, and hypometabolism of, one particular area of the cortex: the ventrolateral anterior temporal lobe (Butler, Brambati, Miller, & Gorno-Tempini, 2009; Mion et al., 2010). Investigations using functional neuroimaging, transcranial magnetic stimulation and intracranial recordings have all confirmed that this region is selectively involved in many forms of verbal and nonverbal semantic processing, as one would expect of a transmodal semantic hub (Humphreys, Hoffman, Visser, Binney, & Lambon Ralph, 2015; Marinkovic et al., 2003; Pobric, Jefferies, & Lambon Ralph, 2007; Shimotake et al., 2015; Visser, Jefferies, Embleton, & Lambon Ralph, 2012).

The hub-and-spoke model, with its commitment to the embodied view that S-M experience plays an important role in shaping semantic representation, provides a parsimonious account of a range of phenomena in normal and impaired semantic processing (Dilkina, McClelland, & Plaut, 2008; Patterson et al., 2007; Lambon Ralph et al., 2017; Rogers et al., 2004; Rogers & McClelland, 2004; Schapiro, McClelland, Welbourne, Rogers, & Lambon Ralph, 2013). Its core principle, that semantic knowledge requires interaction between modality-specific and supramodal levels of representation, is also integral to a number of other theories of semantic cognition (Allport, 1985; Binder & Desai, 2011; Damasio, 1989; Simmons & Barsalou, 2003) and has been employed in other connectionist models (Blouw et al., 2015; Garagnani & Pulvermüller, 2016; Plaut, 2002). There are, however, some critical and challenging aspects of semantic representation which have not been accommodated by these theories, and which we address in this work. First, the representation of abstract concepts is a significant challenge to embodied semantic theories (Binder & Desai, 2011; Leshinskaya & Caramazza, 2016; Meteyard et al., 2012; Shallice & Cooper, 2013). Because abstract words are not strongly linked with S-M experiences, it is unclear how a semantic system based on such experience would represent these concepts. A number of alternative accounts of abstract word knowledge have been put forward, which are not mutually exclusive. First, it is likely that some information about abstract words can be gleaned from the statistics of their use in natural language, an important mechanism that is central to our model and which we will consider in more detail shortly. Second, abstract words often refer to aspects of a person’s internal experiences, such as their emotions or cognitive states (Kousta, Vigliocco, Vinson, Andrews, & Del Campo, 2011; Vigliocco et al., 2014), and it is likely that these internally generated sensations make an important contribution to the representations of some abstract words. These influences were not a specific target of our model, though they are compatible with the approach we take. Finally, it has been suggested that, although abstract words do not represent S-M experiences directly, some abstract words might become grounded in this information through linkage with concrete situations with which they are associated (Barsalou, 1999; Pulvermüller, 2013). For example, the abstract word *direction* might become associated with S-M information related to pointing or to steering a car. However, it remains unclear exactly how abstract words might become associated with S-M experiences. In this study, we make an important advance on this issue by demonstrating how a neural network can learn to associate abstract words with S-M information indirectly, even if its training environment does not include such associations in any direct form.

The representation of associative relationships between items also represents a challenge to embodied semantic models that represent semantic structure in terms of similarity in S-M properties. Such models are highly sensitive to category-based taxonomic structure, because objects from the same taxonomic category (e.g., birds) typically share many S-M characteristics (e.g., have feathers, able to fly; Cree & McRae, 2003; Dilkina & Lambon Ralph, 2012; Garrard, Lambon Ralph, Hodges, & Patterson, 2001). In hub-and-spoke models, for example, as the units in the hub layer learn to mediate between different S-M systems, so objects with similar properties come to be represented by similar patterns of activation (Rogers et al., 2004). However, semantic processing is also strongly influenced by associative relationships between items that are encountered in similar contexts but may have very different properties (e.g., knife and butter; Alario, Segui, & Ferrand, 2000; Lin & Murphy, 2001; Perea & Gotor, 1997; Seidenberg, Waters, Sanders, & Langer, 1984). To represent these relationships, the semantic system must be sensitive to patterns of spatiotemporal co-occurrence among words and objects.

For this reason, some researchers have suggested that taxonomic and associative relations are represented in two distinct systems, rather than a single semantic hub (Binder & Desai, 2011; Mirman & Graziano, 2012; Schwartz et al., 2011). On this view, only the extraction of taxonomic, category-based semantic structure is served by the anterior temporal cortex (ATL). A separate system, linked with ventral parietal cortex (VPC), processes information about actions and temporally extended events and is therefore sensitive to associations between items. An alternative perspective, adopted in the present work, is that both types of relationship are represented within a single semantic space (Jackson, Hoffman, Pobric, & Lambon Ralph, 2015). To do so, the hub must be simultaneously sensitive to similarities in S-M properties and to temporal co-occurrence. As we shall go on to explain in more detail, this can be achieved by training the model to predict upcoming words on the basis of context, in addition to learning the S-M patterns associated with words. Previous computational work by Plaut and colleagues demonstrated that a single semantic system can simulate semantic priming effects for both taxonomic and associative relationships, through sensitivity to item co-occurrence as well as S-M similarity (Plaut, 1995; Plaut & Booth, 2000). That work focused on understanding the timing of access to semantic representations under different conditions. Our focus in the present work is on the structure of the learned semantic representations; we investigate whether the hub-and-spoke architecture develops sensitivity to both types of relationship within a single hub layer.

The final phenomenon we consider is that of context-sensitivity in the processing of meaning. Some words, termed homonyms, take on entirely different meanings when used in different situations (e.g., *bark*). Many more words are polysemous: Their meaning changes in a more subtle and graded fashion across the various contexts in which they appear (consider the change in the meaning of *life* in the two phrases “the mother gave him life” and “the judge gave him life”). While a number of implemented computational models have explored consequences of this ambiguity for lexical processing (Armstrong & Plaut, 2008; Hoffman & Woollams, 2015; Kawamoto, 1993; Rodd, Gaskell, & Marslen-Wilson, 2004), few have considered how context-dependent variation in meaning is acquired or how a contextually appropriate interpretation of a word is activated in any given instance. In order to address such issues, a model must have some mechanism for representing the context in which a particular stimulus is processed. Previous hub-and-spoke models were not developed with this in mind and thus has no such mechanism. Another class of connectionist models have, however, made progress on these issues. Simple recurrent networks process stimuli sequentially and include a buffering function, which allows the network to store the pattern of activity elicited by one input and use this to influence how the next input in the sequence is processed (Elman, 1990). In so doing, simple recurrent networks become highly sensitive to statistical regularities present in temporal streams of information, such as those found in artificial grammars or in sequences of letters taken from English sentences, and can make accurate predictions about upcoming items (Cleeremans, Servan-Schreiber, & McClelland, 1989; Elman, 1990). St. John and McClelland (1990) used a simple recurrent network to represent the meanings of sentences that were presented to the network as a series of individual constituents. Upon processing each constituent, the model was trained to make predictions about the content of the sentence as a whole. Following training, the same word could elicit radically different patterns of activity depending on the particular sentence in which it appeared. This model demonstrated that a simple recurrent network could acquire context-sensitive representations of the meanings of words. The potential value of recurrent networks in developing context-sensitive semantic representations has also been noted by other researchers (Yee & Thompson-Schill, 2016). In the present work, we harness this powerful computational mechanism by integrating it within a hub-and-spoke framework.

To summarize, a number of embodied semantic models hold that concepts are acquired as the semantic system learns to link various verbal and S-M elements of experience through an additional transmodal level of representation. This model is compatible with a range of empirical data but there are three key theoretical issues that remain unresolved. How does such a framework represent the meanings of abstract words? How does it represent associative relations between concepts? And what mechanisms would be necessary to allow its representations to vary depending on the context in which they occur? In tackling these questions, we took inspiration from a different tradition in semantic representation that provides a useful alternative perspective. The *distributional semantics* approach developed in computational linguistics and holds that patterns of lexical co-occurrence in natural language are key determinants of word meanings. Firth (1957) summarized this principle with the phrase “You shall know a word by the company it keeps.” Words that are frequently used in the same or similar contexts are assumed to have related meanings. Modern computing power has allowed this theory to be applied to large corpora of real-world language, with considerable success (Griffiths et al., 2007; Jones & Mewhort, 2007; Landauer & Dumais, 1997; Lund & Burgess, 1996). These statistical models represent words as high-dimensional semantic vectors, in which similarity between the vectors of words is governed by similarity in the contexts in which they are used. Similarity in contextual usage is assumed to indicate similarity in meaning. Representations derived in this way have been shown to be useful in predicting human performance across a range of verbal semantic tasks (Bullinaria & Levy, 2007; Jones & Mewhort, 2007; Landauer & Dumais, 1997).

The distributional semantics approach is well-suited to addressing the challenges in semantic representation we have already identified. Because it is based on linguistic and not S-M experiences, it is possible to code abstract words in exactly the same way as for concrete words. Because its representations are based on contextual co-occurrence, it is highly sensitive to associative relationships between concepts, irrespective of whether they share S-M properties (Hoffman, 2016). Finally, because its central tenet is that meaning is determined by context, it naturally allows for variation in meaning when the same words are used in different contexts (Kintsch, 2001; Landauer, 2001).

The distributional approach has come under heavy criticism because, unlike embodied approaches to semantics, it makes no connection with S-M experiences (Barsalou, 1999; Glenberg & Robertson, 2000). Because the representation of each word is determined solely by its relationships with other words, the system as a whole lacks grounding in the external world. The distributional account would thus seem to provide no insights into the considerable neuroscientific evidence for S-M embodiment of semantic knowledge. Recently, however, some promising efforts have been made to modify distributional models so that they take into account information about S-M properties as well as the statistics of lexical co-occurrence (Andrews et al., 2009; Durda, Buchanan, & Caron, 2009; Johns & Jones, 2012; Steyvers, 2010). These have, for example, shown that S-M properties of concrete words can be accurately inferred by analyzing their patterns of lexical co-occurrence with other words whose S-M characteristics are already known (Johns & Jones, 2012). In addition, a number of researchers have advocated a hybrid view of semantic representation in which embodied and distributional aspects both play a role (Barsalou, Santos, Simmons, & Wilson, 2008; Dove, 2011; Louwerse & Jeuniaux, 2008; Vigliocco, Meteyard, Andrews, & Kousta, 2009). We took a similar position in developing our model.

In the present study, one of our key goals was to develop a connectionist model that combined the distributional approach with the principle of embodiment in S-M experience. Critically, we implemented this synthesis within the hub-and-spoke conceptual framework, which has proved successful in addressing other aspects of semantic representation. In so doing, we addressed another, perhaps more basic limitation of the distributional approach, namely that it provides minimal insights into the mechanisms underpinning acquisition of conceptual knowledge. We will take the most well-known statistical model, latent semantic analysis (Landauer & Dumais, 1997), as an example. This technique involves the construction of a large matrix of word occurrence frequencies, aggregating data from a corpus of several million words. When this matrix has been fully populated, it is subjected to singular value decomposition in order to extract the latent statistical structure thought to underpin semantic knowledge. While the resulting representations appear to bear useful similarities to human semantic knowledge, this process by which they are derived bears little relation to the way in which conceptual knowledge is acquired by humans. Children do not accumulate vast reserves of data about which words they have heard in which contexts, only to convert these data into semantic representations once they have been exposed to several million words. In reality, acquisition of conceptual knowledge is a slow, incremental process, in which knowledge is constantly updated on the basis of new experiences (McClelland, McNaughton, & O’Reilly, 1995). Some researchers have addressed this concern, proposing distributional models in which representations are gradually updated online as linguistic information is processed (Jones & Mewhort, 2007; Rao & Howard, 2008). Nevertheless, the distributional approach to semantic knowledge has yet to be integrated with neurally inspired embodied approaches to semantic cognition.

In this article, we present a model that simultaneously assimilates the embodied and distributional approaches to semantic representation. The basic tenet of the model is that semantic knowledge is acquired as individuals learn to map between the various forms of information, verbal and nonverbal, that are associated with particular concepts (Patterson et al., 2007; Lambon Ralph et al., 2017; Rogers et al., 2004; Rogers & McClelland, 2004). The “hub” that mediates these interactions develops representations that code the deeper, conceptual relationships between items. To this framework, we have added the distributional principle, which holds that sensitivity to context and to the co-occurrence of items is an important additional source of semantic information. To achieve this synthesis, we added two ingredients to the model. The first was a training environment in which concepts are processed sequentially and in which the co-occurrence of concepts in the same sequence is indicative of a semantic relationship between them. The second was a buffering function, inspired by work with simple recurrent networks (Elman, 1990; St. John & McClelland, 1990), that allowed the model’s hub to be influenced by its own previous state. To encourage the model to become sensitive to item co-occurrences, upon processing each stimulus, it was trained to predict the next item in the sequence. This is in tune with the widely held view that prediction is an important mechanism in language processing (Altmann & Kamide, 2007; Dell & Chang, 2013; Pickering & Garrod, 2007) and with recent interest in the use of predictive neural networks to learn distributed representations of word meaning (e.g., Mikolov, Chen, Corrado, & Dean, 2013).

### The Model

#### Overview

**Figure 1 fig1:**
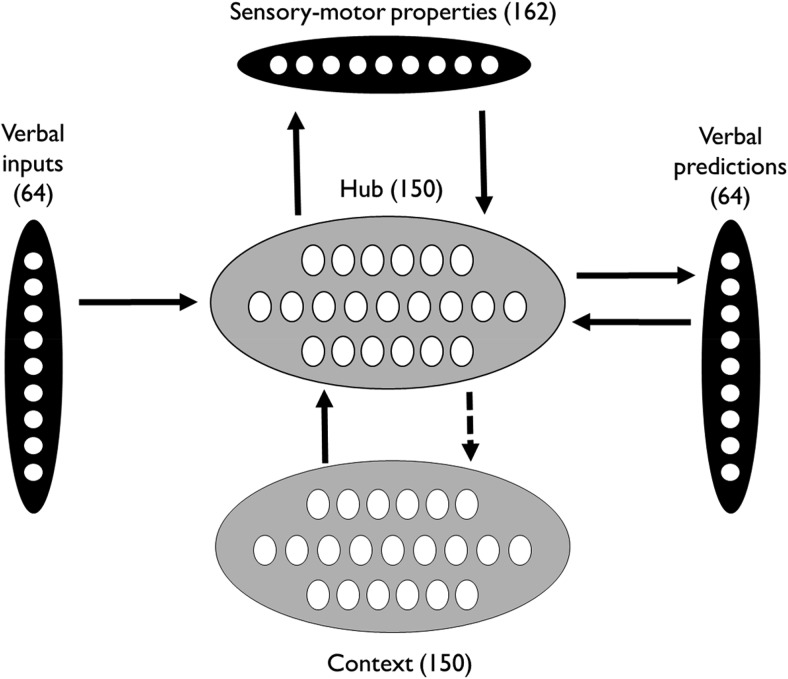
Architecture of the representational model. Black layers comprise visible units that receive inputs and/or targets from the environment. Gray layers represent hidden units. Solid arrows indicate full, trainable connectivity between layers. The dashed arrow represents a copy function whereby, following processing of a stimulus, the activation pattern over the hub layer is replicated on the context layer where it remains to act as the context for the next stimulus.

The model is shown in Figure 1. Inputs are presented to the model sequentially. Inputs may be verbal, analogous to hearing words, or they may be constellations of S-M properties, analogous to interaction with objects in the environment. The model learns to perform two tasks simultaneously in response to these inputs. First, following the presentation of each stimulus, it is required to make predictions about which word will appear next in the sequence, taking into account recent context. Second, when presented with a concrete word as a stimulus, it is also required to activate the S-M properties of the word’s referent.[Fig-anchor fig1]

#### Architecture

The model is a fully recurrent neural network, consisting of 590 units organized into five pools. Sixty-four verbal input units represent the 64 words in the model’s vocabulary. Activation of these units is controlled by external input from the environment. In contrast, the 64 verbal prediction units never receive external inputs, but are used to represent the model’s predictions about the identity of the next word in the sequence. There are 162 units representing S-M properties. These can either activated externally, representing perception of an object in the environment, or they can be activated by the model in the course of processing a particular verbal input. This latter process can be thought of as a mental simulation of the properties of an object upon hearing its name.

The connections between the three layers are mediated by 150 hidden units, known collectively as the “hub.” Activation patterns over the hub layer are not specified directly by the modeler and are instead shaped by the learning process. As the hub is trained to map between verbal inputs, verbal predictions and S-M properties, it develops patterns of activation that reflect the statistics underlying these mappings. Words that are associated with similar verbal predictions and/or similar S-M properties come to be represented by similar activation patterns in the hub.

Finally, at each step in the sequence, the 150 context units are used to store a copy of the hub activations elicited by the previous input (see Processing section for more detail). This information is an additional source of constraint on the hub, allowing its processing of each input to be influenced by the context in which it occurs.

#### Processing

**Figure 2 fig2:**
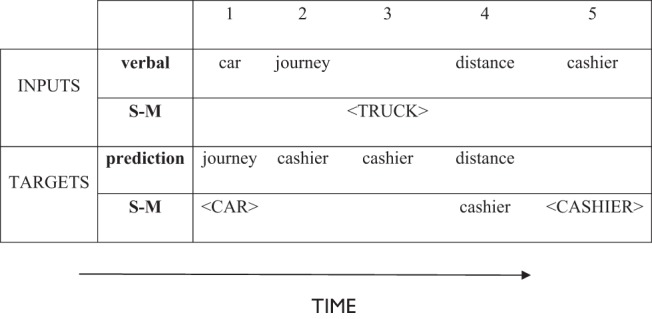
An example episode. The 10 inputs for the episode are shown from left to right, along with the targets provided at each point. For example, at the first point in this sequence, the verbal input unit for *car* is activated and the model is trained to turn on the S-M units associated with cars and the prediction unit for *journey* (as this is the next item in the sequence). <ITEM > represents the S-M properties of a concrete item.

The model is presented with sequences of stimuli consisting of words and S-M properties, arranged in “episodes” of five inputs. An example episode is shown in Figure 2. As we were primarily concerned with comprehension of individual words, sequences have no syntactic structure and consist entirely of nouns. The word sequences therefore do not represent sentences as such; instead, they represent a series of concepts that one might encounter while listening to a description of an event or a scene. At some points in the sequence, a set of S-M properties representing a particular concrete object is presented in lieu of a word. This reflects the fact that when we are listening to a verbal statement, we often simultaneously observe objects in the environment that are relevant to the topic under discussion. In the model, this concurrent experience of verbal and nonverbal stimuli is implemented as a sequential process, with the nonverbal perceptions interspersed within the verbal stream.[Fig-anchor fig2]

Each stimulus is processed for seven time steps, with unit activations updated four times in each time step. To present the model with a word, the corresponding verbal input unit is clamped on for the full seven time steps and activation is allowed to propagate through the rest of the network. No direct input is provided to the prediction or S-M units; instead, their activity develops as a consequence of the flow of activation through the network in response to the word. At the end of this process, the activation states of the prediction and S-M units can be read off as the model’s outputs. Once fully trained, the model produces a pattern of activation over its prediction units that represents its expectation about the identity of the next word, given the word just presented and the preceding context. Activation of S-M units represents the S-M properties that the model has come to associate with the presented word.

During the training phase, the model is presented with targets that are used to influence learning. During the final two time steps for each stimulus, it receives targets on the prediction layer and, optionally, on the S-M layer. The prediction unit representing the next word in the episode is given a target value of one (all other prediction units have targets of zero). When the input is a concrete word or homonym, the model is also given S-M targets corresponding to the S-M properties of the word’s referent. If the input is an abstract word, no S-M targets are provided and the model is free to produce any pattern of activity over the S-M units. The actual activation patterns over the prediction and S-M layers are compared with their targets so that errors can be calculated and the connection weights throughout the network adjusted by back-propagation (see training and other model parameters). When abstract words are presented, there are no targets on the S-M units.

When the model is presented with a S-M pattern as stimulus, the process is similar. The S-M units are clamped for the full seven time steps and the verbal input units are clamped at zero. Activation propagates through the network and targets are provided for the prediction layer during the final two time steps. The prediction target again represents the next word in the episode.

Following the processing of each stimulus, the activation values of the hub units are copied over to the context units. The context units are then clamped with this activation pattern for the duration of the next stimulus. The context units provide an additional input to the hub layer, allowing it to be influenced by its previous state. This recurrent architecture allows the model to develop representations that are sensitive to context.

#### Model vocabulary

**Figure 3 fig3:**
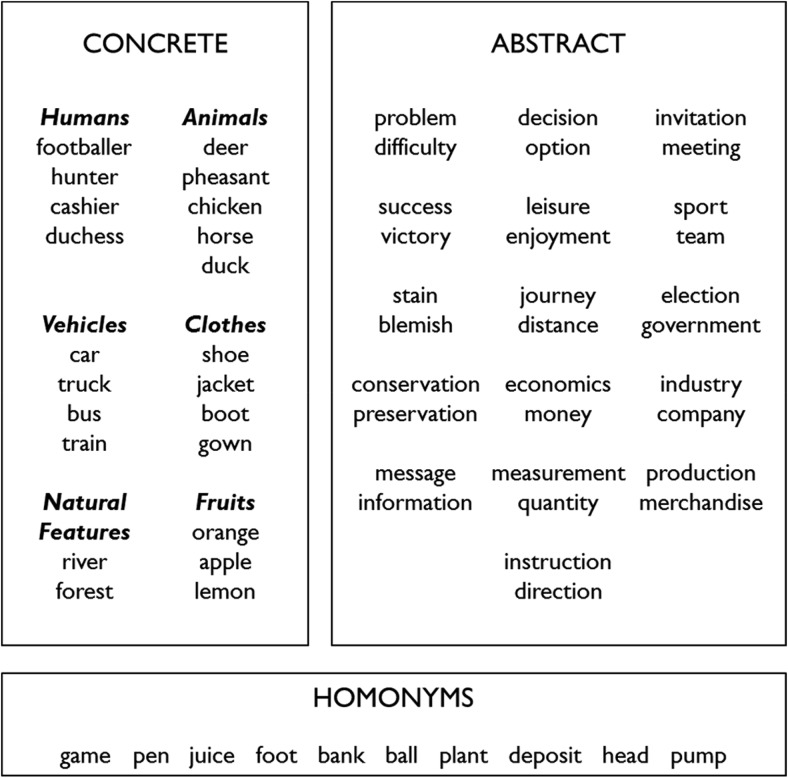
The model’s vocabulary.

In common with other connectionist approaches to semantics, the model was trained in a simplified artificial environment designed to capture the key features of semantic processing that are relevant to our goals. The 64 concepts in the model’s vocabulary comprise 22 concrete concepts, 32 abstract concepts, and 10 homonyms (see Figure 3). The concrete and abstract words were used to investigate how knowledge for abstract concepts could become embodied in S-M experience (see below and Simulation 2). The concrete words were also used to explore the model’s ability to represent taxonomic and associative semantic relationships (Simulation 3). The homonyms, which we define as words that have two meanings associated with distinct contexts, were used to investigate the model’s sensitivity to context (Simulation 1).[Fig-anchor fig3]

#### S-M properties

The 162 S-M units represent the sensory and motor properties of objects. Many studies have investigated how the structure of S-M properties varies across different categories of object (e.g., Cree & McRae, 2003; Garrard et al., 2001) and insights from these studies have been incorporated into models that seek to explain dissociations between particular categories (e.g., Farah & McClelland, 1991; Tyler, Moss, Durrant-Peatfield, & Levy, 2000). Such effects were not germane in the present study so we only implemented the most robust general finding in this domain: that members of the same category tend to share more S-M properties than items from different categories. Concrete concepts were organized into six taxonomic categories (see Figure 3). Each item was associated with six properties that it shared with its category neighbors and three that were unique to that item. Abstract concepts were not assigned S-M properties, on the basis that these concepts are not linked directly with specific S-M experiences. In natural language, the meanings of homonyms can be either concrete or abstract. In the model, we assumed for simplicity that all homonyms had concrete meanings.^1^ We assigned two different sets of S-M properties to each homonym, corresponding to each of its meanings. Each set consisted of six properties shared with other concrete concepts and three properties unique to that meaning.

#### Training corpus

**Figure 4 fig4:**
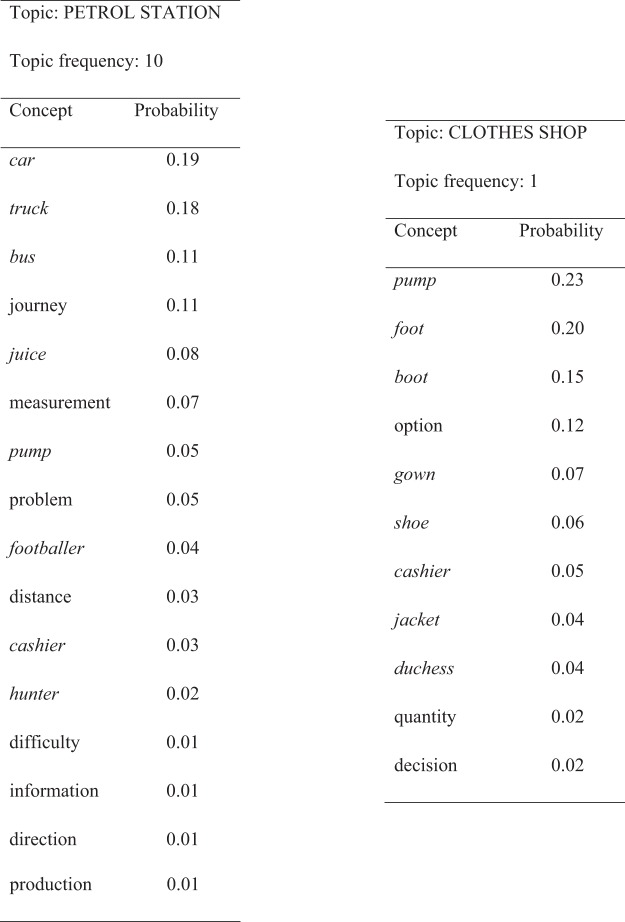
Example topic distributions. Concepts with S-M features are shown in italics. The PETROL STATION topic was used to generate the episode shown in Figure 2.

Our construction of a training corpus for the model was inspired by a particular class of distributional semantic models known as topic models (Griffiths et al., 2007). These models assume that samples of natural language can be usefully represented in terms of underlying topics, where a topic is a probability distribution over a particular set of semantically related words. To generate a training corpus for our model, we constructed 35 artificial topics. An example topic is shown in Figure 4. Each topic consisted of a list of between 10 and 19 concepts that might be expected to be used together in a particular context. There was also a probability distribution that governed their selection. The construction of topics was guided by the following constraints:

1 Topics were composed of a mixture of concrete, abstract and homonym concepts (although two topics, ELECTION and REFERENDUM, featured only abstract concepts).[Fig-anchor fig4]
2 Abstract concepts were organized in pairs with related meanings (see Figure 3). Word pairs with related meanings frequently occurred in the same topics, in line with the distributional principle. That is, words with related meanings had similar (but not identical) probability distributions across the 35 topics. For example, *journey* and *distance* could co-occur in seven different topics, but with differing probabilities, and there were an additional five topics in which one member of the pair could occur but the other could not.
3 Concrete concepts belonging to the same category frequently occurred in the same topics, in line with distributional data from linguistic corpora (Hoffman, 2016) and visual scenes (Sadeghi, McClelland, & Hoffman, 2015). In addition, particular pairs of concrete concepts from different categories co-occurred regularly in specific topics (e.g., *deer* and *hunter* both appeared with high probability in the HUNTING topic). This ensured that the corpus included associative relationships between items that did not share S-M properties.
4 Each homonym occurred in two disparate sets of topics. For example, *bank* regularly occurred in the FINANCIAL topic, representing its dominant usage, but also occasionally in the RIVERSIDE topic, representing its subordinate meaning.^2^

Some additional constraints, required for Simulation 2, were also included and are described as they become relevant.

The topics were used to generate episodes consisting of 5 stimuli. To generate an episode, a topic was first chosen in a stochastic fashion, weighted such that eight particular topics were selected five or 10 times more often than the others. This weighting ensured that some concepts occurred more frequently than others (necessary for Simulation 2). Next, a concept was sampled from the probability distribution for the chosen topic. If a concrete concept or homonym was chosen, it was presented either verbally or as a S-M pattern (with equal probability). For concrete words, the S-M pattern used was always the same. For homonyms, the S-M pattern varied depending on whether the word was being used in its dominant or subordinate sense. Another concept was then sampled and the process continued until a sequence of five stimuli had been generated. The same concept could be sampled multiple times within an episode.

A total of 400,000 episodes were generated in this fashion; this served as the training corpus for the model. The corpus was presented as a continuous stream of inputs to the model, so there was no indication of when one episode ended and the next began. On the last stimulus for each episode, however, no prediction target was given to the model.

#### Training and other model parameters

Simulations were performed using the Light Efficient Network Simulator (Rohde, 1999). The network was initialized with random weights that varied between −0.2 and 0.2. All units were assigned a fixed, untrainable bias of −2, ensuring that they remained close to their minimum activation level in the absence of other inputs. Activation of the hub units and S-M units was calculated using a logistic function. Error on the S-M units was computed using a cross-entropy function. As the prediction units represented a probability distribution, their activation was governed by a soft-max function which ensured that their combined activity always summed to one. These units received a divergence error function.

The model was trained with a learning rate of 0.1 and momentum of 0.9, with the condition that the premomentum weight step vector was bounded so that its length could not exceed one (known as “Doug’s momentum”). Error derivatives were accumulated over stimuli and weight changes applied after every hundredth episode. Weight decay of 10^−6^ was applied at every update. The model was trained for a total of five passes through the corpus (equivalent to 20,000 weight updates, two million episodes, or 10 million individual stimuli).

Ten models were trained in this way, each with a different set of random starting weights. All the results we present are averaged over the 10 models.

### Results: Representational Properties of the Model

#### Context-sensitivity

**Figure 5 fig5:**
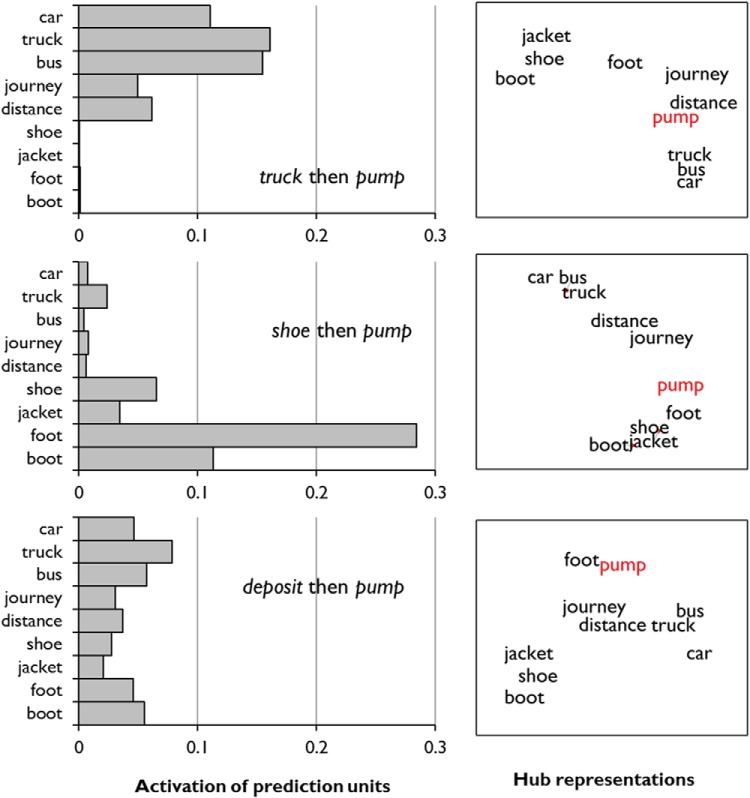
Context-sensitive representation of the word *pump.* The model was presented with *pump* immediately following either *truck*, *shoe*, or *deposit*. Results are averaged over 50 such presentations. Left: Activation of prediction units, indicating that the model’s expectations change when the word appears in these different contexts. Right: Results of multidimensional scaling analyses performed on the hub representations of words presented in each context. In these plots, the proximity of two words indicates the similarity of their representations over the hub units (where similarity is measured by the correlation between their activation vectors). The model’s internal representation of *pump* shifts as a function of context.

Once trained, the model is able to take a word as input and predict which other words it is likely to encounter subsequently. Due to its recurrent architecture, these predictions are shaped by the context in which the word is presented. To illustrate this, we presented the word *pump* to the model immediately after one of three other words. Two of these words, *truck* and *shoe*, represent the two disparate types of context in which *pump* appeared during training. The third, *deposit* represents a novel context. The left-hand panel of Figure 5 shows activation of some of the network’s prediction units in each context. The model demonstrates context-sensitivity, appropriately biasing its predictions toward petrol-related words in the first case and clothing-related words in the second. When the word appears in a novel context, the model hedges its bets and assigns intermediate probabilities to both types of word. [Fig-anchor fig5]

The model is able to shift its behavior in this way because the learned representations over the hub layer are influenced by prior context. This is illustrated in the right-hand panel of Figure 5, which represents graphically the relationships between the network’s representations of particular words in the three different contexts. We presented the network with various words, each time immediately after one of the three context words, and recorded the pattern of activity over the hub units. We performed multidimensional scaling on these representations, so that each word could be plotted in a two-dimensional space in which the proximities of words indicates the degree of similarity in their hub representations. When presented in the context of *truck*, the model’s representation of *pump* is similar to that of *journey*, *distance* and other petrol-related words. Conversely, when *pump* is presented after *shoe*, the model generates an internal representation that is similar to that of *foot* and other items of clothing. In a novel context, the *pump* representation lies in the midst of these two sets. In other words, by including context units that retain the network’s previous states, the model has developed semantic representations for words that take into account the context in which they are being used. This context-dependence is a key feature of models with similar recurrent architectures (Elman, 1990; St. John & McClelland, 1990).

It is worth noting that these context-dependent shifts in representation are graded and not categorical. In other words, the model’s representation of a word’s meaning varies continuously as a function of the context in which it is being used. This graded variation in representation is consistent with a proposition from the distributional semantics approach, which holds that any two uses of the same word are never truly identical in meaning. Instead, their precise connotation depends on their immediate linguistic and environmental context (Cruse, 1986; Landauer, 2001). This means that, in addition to homonyms, the model is well-suited to the representation of polysemous words, whose meanings change more subtly when they are used in different contexts. We consider this aspect of the model in more detail in Simulation 2, where we simulate the effects of semantic diversity on comprehension (Hoffman, Lambon Ralph, & Rogers, 2013b; Hoffman, Rogers, & Lambon Ralph, 2011b).

#### Representation of abstract words and taxonomic and associative semantic structure

**Figure 6 fig6:**
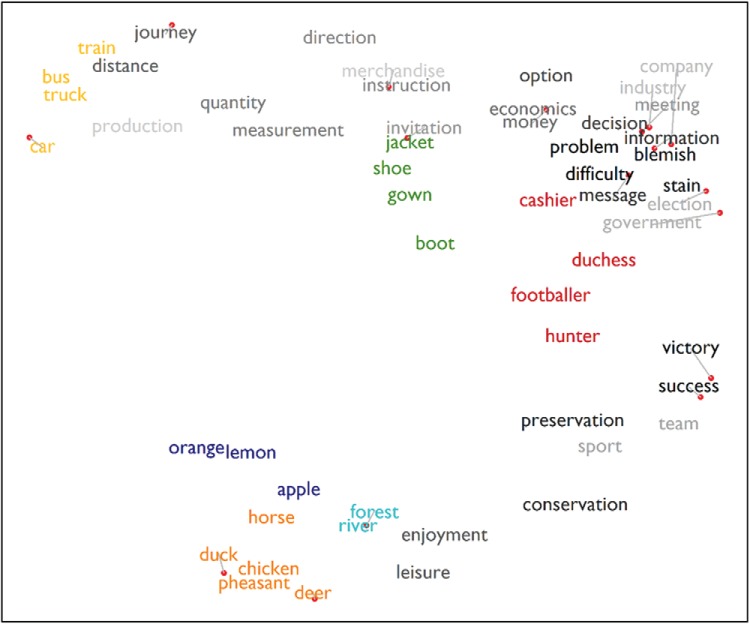
Hub representations of concrete and abstract concepts. Concrete concepts are color-coded by category. Abstract concepts are shown in greyscale, where shading indicates pairs of semantically related words.

A key feature of the model is that all concepts, concrete and abstract, are associated with characteristic patterns of activity over the same hub units and are therefore represented in a common semantic space. To explore the characteristics of this space, we performed multidimensional scaling on the hub’s representations of all concrete and abstract words. In this case, we were interested in the general structure of the semantic space, independent of any specific context. We therefore presented each word to the network 64 times, each time preceded by a different word from the model’s vocabulary. To obtain context-independent representations for each word, we averaged the activation patterns elicited on the hub units over these 64 presentations. The resulting activation patterns for all words were used to compute a pairwise distance matrix between words. The process was repeated 50 times and the averaged distance matrix was used to generate the multidimensional scaling plot shown in Figure 6.[Fig-anchor fig6]

The model acquires internal representations that allow it to generate appropriate patterns of activity over the S-M and prediction units. As a consequence, words that are associated with similar S-M features come to be associated with similar hub representations, as do those that elicit similar predictions about upcoming words. Several consequences of this behavior are evident in Figure 6.

1 Taxonomic structure emerges as an important organizational principle for concrete words. There are two reasons why the model learns this representational structure. First, concrete items from the same category share a number of S-M features. Second, items from the same category regularly occur in the same contexts and are therefore associated with similar predictions about which words are likely to appear next.
2 Abstract words that occur in similar contexts have similar representations. The corpus was designed such that particular pairs of abstract words frequently co-occurred (see Figure 2). In Figure 6, it is clear that these pairs are typically close to one another in the network’s learned semantic space. When the model is presented with abstract words, it is only required to generate predictions; therefore, the representation of abstract words is governed by the distributional principle. Words that frequently occur in the same contexts come to have similar semantic representations because they generate similar predictions.
3 The units in the hub make no strong distinction between concrete and abstract words. Concrete and abstract words can be represented as similar to one another if they occur in similar contexts (e.g., *journey* and *distance* and the vehicles). Of course, concrete and abstract words are more strongly distinguished in the S-M units, where only concrete words elicit strong patterns of activity (though abstract words come to generate some weaker activity here too; see below).
4 Associative relationships between concrete items are also represented. Although taxonomic category appears to be the primary organizing factor for concrete concepts, the structure of these items also reflects conceptual co-occurrence. For example, the fruits, plants, and animals are all close to one another because they regularly co-occur in contexts relating to the outdoors/countryside (in addition, some of the animals and fruits co-occur in cooking contexts).

To investigate the degree to which the model acquired associative as well as taxonomic relationships, we performed further analyses on pairwise similarities between the hub representations of the concrete items. The mean similarity between item pairs from the same category was 0.44 (*SD* = .061) while for between-category pairs it was 0.01 (*SD* = .056; *t*(229) = 40.5, *p* < .001). This confirms our assertion that items from the same category have much more similar representations than those from different categories. To investigate the effect of associative strength on representational similarity, we considered the between-category pairs in more detail. We defined the associative strength *A* between two words *x* and *y* as follows:

$$A=\frac{1}{2}\left(\frac{N_{xy}}{N_{x}}+\frac{N_{yx}}{N_{y}}\right)$$

Where *N*_*xy*_ indicates the number of occasions *x* was immediately followed by *y* in the training corpus, *N*_*yx*_ is the number of times *y* was followed by *x* and *N*_*x*_ and *N*_*y*_ represent the total number of occurrences of *x* and *y*, respectively. There was a significant positive correlation between the associative strength of two items and the similarity of their hub representations, ρ(199) = 0.39, *p* < .001. In other words, the more frequently two items occur together during training, the more likely the model is to represent them with similar patterns in the hub. The average similarity for strongly associated between-category pairs (defined with an arbitrary threshold of *A* > 0.07) was 0.10 (*SD* = .08).

#### Acquired embodiment of abstract concepts

**Figure 7 fig7:**
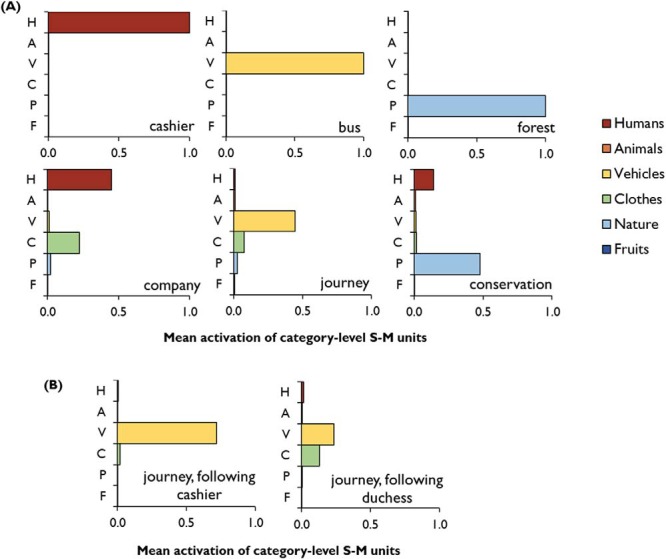
S-M unit activations for a selection of concrete and abstract words. (A) Activations of S-M units shared by the members of each category, in response to a selection of words. Each word was presented to the network 50 times (with a different random pattern of activity on the context units) and the results averaged to generate this figure. (B) Activation of S-M units in response to the same abstract word in two different contexts.

As discussed earlier, the representation of abstract concepts is a contentious issue. Some researchers have suggested that knowledge of abstract words is derived solely through their use in language. Others have argued that abstract concepts must be grounded in perceptual experience (e.g., Barsalou, 1999) but it is not clear how such grounding would take place. When being trained to process abstract words, our model only receives verbal distributional information; it is not trained to associate abstract words with S-M experiences. However, abstract words come to be linked to S-M information by virtue of their associations with concrete words—a process we refer to as “acquired embodiment.” Figure 7A provides some examples of this. We have plotted activations for the S-M features shared by all members of a category when the network is presented with some representative concrete and abstract words. For concrete words, the network is trained to activate the S-M features of the item whenever it is encountered. Each of the concrete words therefore elicits a clear, binary pattern of S-M activation. For abstract words, the S-M units do not receive any targets during training, in line with the idea that abstract concepts are not *directly* associated with S-M experiences. The activity of these units is entirely unconstrained by the learning process. As seen in Figure 7A, however, when presented with abstract words, the network comes to partially activate the S-M features of the concrete items with which they regularly co-occur. For example, *journey* elicits partial activation of the S-M features of vehicles and *company* partially activates the features of humans. [Fig-anchor fig7]

This acquired embodiment is an emergent consequence of the requirement for the model to represent the statistics of conceptual co-occurrence and S-M experience in a single system. As we saw earlier, the model represents concrete and abstract words in a single semantic space and both can elicit similar patterns of activity on the hub layer if they are associated with similar verbal predictions. For example, *journey* has a similar representation to *bus* because both words are found in contexts in which words like *car, distance*, and *pump* are likely to occur. Because the activity of the S-M units is determined by the inputs they receive from the hub units, words with similar hub representations generate similar patterns of S-M activity. So *journey* comes to partially activate vehicular S-M features as a by-product of its regular co-occurrence with vehicle names.

A number of alternative modeling approaches have also merged S-M information with distributional statistics from natural language (Andrews et al., 2009; Durda et al., 2009; Steyvers, 2010) and have shown how S-M knowledge linked with a particular word can be indirectly extended to its lexical associates (Johns & Jones, 2012). One important way in which our model differs from these other approaches is that, in our model, the embodiment of abstract words is context-dependent. This is illustrated in Figure 7B, which shows the different S-M activations elicited by the same abstract words in two different contexts. When *journey* occurs immediately after *cashier,* vehicle S-M units are strongly activated because *journey* and *cashier* regularly co-occur in contexts in which modes of transport are discussed. In contrast, *journey* presented after *duchess* elicits only weak activation because in the topics in which these two words co-occur, vehicles are rarely. Thus, the type of S-M information activated by abstract words depends on the particular context in which they appear, which is consistent with data showing that context affects the types of S-M knowledge participants retrieve in response to words (Wu & Barsalou, 2009).

### Summary

In this section, we have described how our model acquires semantic representations under the simultaneous pressure to predict upcoming words based on preceding context (thus learning the distributional properties of the language) and to associate concrete words with S-M experiences (thus embodying conceptual knowledge in the physical world). Importantly, both of these challenges are met by a single set of “hub” units, whose activation patterns come to represent the underlying semantic structure of the concepts processed by the model. We have demonstrated that this architecture has a number of desirable characteristics. The recurrent architecture allows the network’s predictions about upcoming words to be influenced by prior context. As a consequence, the model’s internal representations of specific concepts also vary with context. This is an important property, because most words are associated with context-dependent variation in meaning (Cruse, 1986; Klein & Murphy, 2001; Rodd, Gaskell, & Marslen-Wilson, 2002). Second, the model represents concrete and abstract words in a single representational space, and is sensitive to associative semantic relationships as well as those based on similarity in S-M features. This is consistent with neuroimaging and neuropsychological evidence indicating that all of these aspects of semantic knowledge are supported by the transmodal “hub” cortex of the ventral anterior temporal lobes (Hoffman, Binney, & Lambon Ralph, 2015; Hoffman, Jones, & Lambon Ralph, 2013a; Jackson et al., 2015; Jefferies, Patterson, Jones, & Lambon Ralph, 2009). Finally, the model provides an explicit account of how abstract words can become indirectly associated with S-M information by virtue of their co-occurrence with concrete words. This process of acquired embodiment demonstrates how representations of abstract words based on the distributional principle can become grounded in the physical world.

At the outset of this article, we stated that a comprehensive theory of semantic cognition requires not only an account of how semantic knowledge is represented but also how it is harnessed to generate task-appropriate behavior. In the next section, we turn our attention to this second major challenge: The need for control processes that regulate how semantic information is activated to complete specific tasks.

## Part 2: Executive Regulation of Semantic Knowledge

The semantic system holds a great deal of information about any particular concept and different aspects of this knowledge are relevant in different situations. Effective use of semantic knowledge therefore requires that activation of semantic knowledge is shaped and regulated such that the most useful representation for the current situation comes to mind. An oft-quoted example is the knowledge required to perform different tasks with a piano (Saffran, 2000). When *playing* a piano, the functions of the key and pedals are highly relevant and must be activated in order to guide behavior. However, when *moving* a piano, this information is no longer relevant and, instead, behavior should be guided by the knowledge that pianos are heavy, expensive and often have wheels. The meanings of homonyms are another case that is germane to the present work. When a homonym is processed, its distinct meanings initially compete with one another for activation and this competition is thought to be resolved by top-down executive control processes, particularly when context does not provide a good guide to the appropriate interpretation (Noonan, Jefferies, Corbett, & Lambon Ralph, 2010; Rodd, Davis, & Johnsrude, 2005; Zempleni, Renken, Hoeks, Hoogduin, & Stowe, 2007).

These top-down regulatory influences are often referred to as *semantic control* (Badre & Wagner, 2002; Jefferies & Lambon Ralph, 2006) and are associated with activity in a neural network including left inferior frontal gyrus, inferior parietal sulcus and posterior middle temporal gyrus (Badre, Poldrack, Paré-Blagoev, Insler, & Wagner, 2005; Bedny, McGill, & Thompson-Schill, 2008; Noonan, Jefferies, Visser, & Lambon Ralph, 2013; Rodd et al., 2005; Thompson-Schill et al., 1997; Whitney, Kirk, O’Sullivan, Lambon Ralph, & Jefferies, 2011a, 2011b; Zempleni et al., 2007). One long-standing source of evidence for the importance of semantic control comes from stroke patients who display semantic deficits following damage to these areas (Harvey, Wei, Ellmore, Hamilton, & Schnur, 2013; Jefferies, 2013; Jefferies & Lambon Ralph, 2006; Noonan et al., 2010; Schnur, Schwartz, Brecher, & Hodgson, 2006). These patients, often termed *semantic aphasics* (SA, after Head, 1926), present with multimodal semantic impairments but, unlike the SD patients described earlier, their deficits have been linked with deregulated access to semantic knowledge rather than damage to the semantic store itself. Moreover, these patients’ performance on semantic tasks is strongly influenced by the degree to which the task requires executive regulation and the severity of their semantic impairments is correlated with their deficits on nonsemantic tests of executive function (which is not the case in SD; Jefferies & Lambon Ralph, 2006). Indeed, there is ongoing debate as to the degree to which semantic control recruits shared executive resources involved in other controlled processing in other domains (we consider this in the General Discussion).

Semantic control deficits have been linked with the following problems.

1 *Difficulty tailoring activation of semantic knowledge to the task at hand.* This is evident in picture naming tasks, in which SA patients frequently give responses that are semantically associated with the pictured object but are not its name (e.g., saying “nuts” when asked to name a picture of a squirrel; Jefferies & Lambon Ralph, 2006). In category fluency tasks, patients are also prone to name items from outside the category being probed (Rogers et al., 2015).
2 *Difficulty selecting among competing semantic representations.* SA patients perform poorly on semantic tasks that require selection among competing responses, particularly when the most obvious or prepotent response is not the correct one (Jefferies & Lambon Ralph, 2006; Thompson-Schill et al., 1998). This problem is also evident in the “refractory access” effects exhibited by this group, in which performance deteriorates when competition between representations is increased by presenting a small set of semantically related items rapidly and repeatedly (Jefferies, Baker, Doran, & Lambon Ralph, 2007; Warrington & Cipolotti, 1996). These deficits are thought to reflect impairment in executive response selection mechanisms.
3 *Difficulty identifying weak or noncanonical semantic associations.* SA patients find it difficult to identify weaker semantic links between concepts (they can identify necklace and bracelet as semantically related but not necklace and trousers; Noonan et al., 2010). They have difficulty activating the less frequent meanings of homonyms (see Simulation 1). In the nonverbal domain, SA patients have difficulty selecting an appropriate object to perform a task when the canonical tool is unavailable (e.g., using a newspaper to kill a fly in the absence of a fly swat; Corbett, Jefferies, & Lambon Ralph, 2011). These results may indicate deficits in top-down “controlled retrieval” processes that regulate semantic activation in the absence of strong stimulus-driven activity (see below).
4 *High sensitivity to contextual cues.* Performance on verbal and nonverbal semantic tasks improves markedly when patients are provided with external cues that boost bottom-up activation of the correct information, thus reducing the need for top-down control (Corbett et al., 2011; Hoffman, Jefferies, & Lambon Ralph, 2010; Jefferies, Patterson, & Lambon Ralph, 2008; Soni et al., 2009). For example, their comprehension of the less common meanings of homonyms (e.g., *bank-river*) improves when they are provided with a sentence that biases activation toward the appropriate aspect of their meaning (e.g., “They strolled along the bank;” Noonan et al., 2010). These findings indicate that these individuals retain the semantic representations needed to perform the task but lack the control processes necessary to activate them appropriately.

Despite the importance of control processes in regulating semantic activity, this aspect of semantic cognition has rarely been addressed in computational models. Where efforts have been made, these have been based on the “guided activation” approach to cognitive control (Botvinick & Cohen, 2014). On this approach, representations of the current goal or task, often assumed to be generated in prefrontal cortex, bias activation elsewhere in the system to ensure task-appropriate behavior. The best-known example of this approach is the connectionist account of the Stroop effect, in which task units represent the goals “name word” and “name color” and these potentiate activity in the rest of the network, constraining it to produce the appropriate response on each trial (Cohen, Dunbar, & McClelland, 1990). In the semantic domain, models with hub-and-spoke architectures have used task units to regulate the degree to which different spoke layers participate in the completion of particular tasks (Dilkina et al., 2008; Plaut, 2002). Although semantic control was not the focus of these models, they do provide a plausible mechanism by which control could be exercised in situations where the task-relevant information is signaled by an explicit cue. For example, one task known to have high semantic control demands is the feature selection task, in which participants are instructed to match items based on a specific attribute (e.g., their color) while ignoring other associations (e.g., *salt* goes with *snow*, not *pepper*; Thompson-Schill et al., 1997). SA patients have great difficulty performing this task (Thompson, 2012) and it generates prefrontal activation in a region strongly associated with semantic control (Badre et al., 2005). To simulate performance on this task in a hub-and-spoke architecture, a task representation could be used to bias activation toward units representing color and away from other attributes, thus biasing the decision-making process toward the relevant information for the task and avoiding the prepotent association.

In the present study, we consider a different aspect of semantic control which, to our knowledge, has yet to receive any attention in the modeling literature. It is well-known that detecting weak semantic associations (e.g., bee-pollen), compared with strong ones (bee-honey), activates frontoparietal regions linked with semantic control (Badre et al., 2005; Wagner, Paré-Blagoev, Clark, & Poldrack, 2001). SA patients with damage to the semantic control network also exhibit disproportionately severe deficits in identifying weak associations (Noonan et al., 2010). However, the cognitive demands of this task are rather different to the ones described in the previous paragraph. In the Stroop and feature selection tasks, participants are instructed to avoid a prepotent response option in favor of a less obvious but task-appropriate response. But in the weak association case, the difficulty arises from the fact that *none* of the response options has a strong, prepotent association with the probe word. For example, a participant may be asked whether *bee* is more strongly associated with *knife, sand*, or *pollen.* When one thinks of the concept of a *bee*, one may automatically bring to mind their most common properties, such as buzzing, flying, making honey, and living in hives. Because these dominant associations do not include any of the response options, the correct answer can only be inferred by activating the bees’ less salient role in pollinating flowers.

In this situation, when automatic, bottom-up processing of the stimuli has failed to identify the correct response, it has been proposed that participants engage in a top-down “controlled retrieval” process (Badre & Wagner, 2002; Gold & Buckner, 2002; Wagner et al., 2001; Whitney et al., 2011b). Badre and Wagner (2002) describe this process as follows:

Controlled semantic retrieval occurs when representations brought online through automatic means are insufficient to meet task demands or when some prior expectancy biases activation of certain conceptual representations. Hence, controlled semantic retrieval may depend on a top-down bias mechanism that has a representation of the task context, either in the form of a task goal or some expectancy and that facilitates processing of task-relevant information when that information is not available through more automatic means. (p. 207)

Although various authors have discussed the notion of a controlled retrieval mechanism for supporting the detection of weak associations, no attempts have been made to specify how such a process would actually operate. This is, we believe, a nontrivial issue. Task representations of the kind described earlier are unlikely to be helpful since the task instruction (“decide which option is most associated with this word”) provides no clue as to what aspect of the meaning of the stimulus will be relevant. In some cases, prior semantic context may provide a useful guide (e.g., the bee-pollen association may be detected more easily if one is first primed by reading “the bee landed on the flower”). Indeed, Cohen and Servan-Schreiber (1992) proposed a framework for cognitive control in which deficits in controlled processing stemmed from an inability to maintain internal representations of context. The same mechanism was used to maintain task context in the Stroop task and to maintain sentence context in a comprehension task. For these researchers, then, the role of top-down control in semantic tasks was to maintain a representation of prior context that can guide meaning selection. However, in most of the experiments that have investigated controlled retrieval, no contextual information was available and thus this account is not applicable. Furthermore, as we have stated, SA patients show strong positive effects of context, which suggests that an inability to maintain context representations is not the source of control deficits in this group.

How, then, do control processes influence activity in the semantic network in order to detect weak relationships between concepts? In the next section, we address this issue by describing an explicit mechanism for controlled retrieval in our model. The core assumption of our approach is that, in order to reach an appropriate activation state that codes the relevant semantic information, the semantic system must be simultaneously sensitive to the word being probed and to its possible associates. Controlled retrieval takes the form of a top-down mechanism that forces the network to be influenced by all of this information as it settles, and which iteratively adjusts the influence of each potential associate. In so doing, the network is able to discover an activation state that accommodates both the probe and the correct associate.

### Controlled Retrieval of Semantic Information

**Figure 8 fig8:**
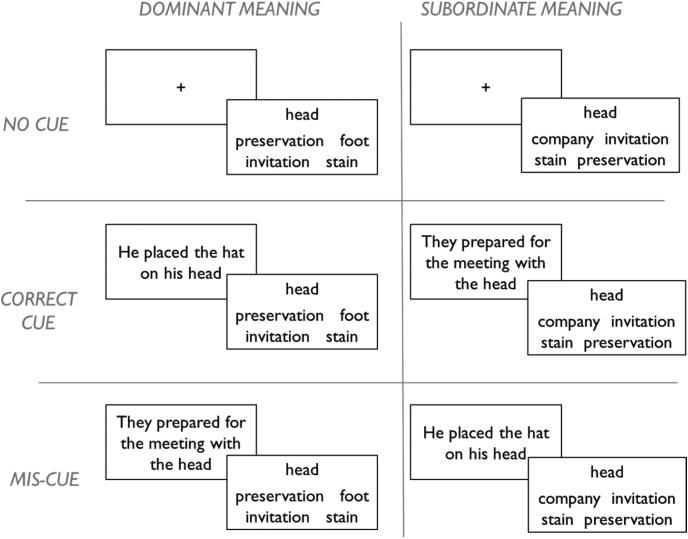
Example trials from the homonym comprehension task.

To illustrate the controlled retrieval process, we need to introduce an experimental task (Noonan et al., 2010) that will later form the basis for Simulation 1. Figure 8 shows some example stimuli. The experiment probes comprehension of homonyms using a 2 (meaning dominance) × 3 (context) design. On each trial, participants are presented with a probe (*head* in Figure 8) and asked to select which of four alternatives has the strongest semantic relationship with it. Half of the trials probe the dominant meanings of the homonyms (e.g., head-foot) and half their subordinate meanings (head-company). The subordinate trials represent a case in which controlled retrieval is thought to be key in identifying the correct response, because bottom-up semantic activation in response to the probe will tend toward its dominant meaning. Furthermore, each trial can be preceded by one of three types of context: either a sentence that primes the relevant meaning of the word (correct cue), a sentence that primes the opposing meaning (miscue), or no sentence at all (no cue). These conditions are randomly intermixed throughout the task so that participants are not aware whether the cue they receive on each trial is helpful or not. The context manipulation allows us to explore how external cues can bias semantic processing toward or away from aspects of meaning relevant to the task. [Fig-anchor fig8]

**Figure 9 fig9:**
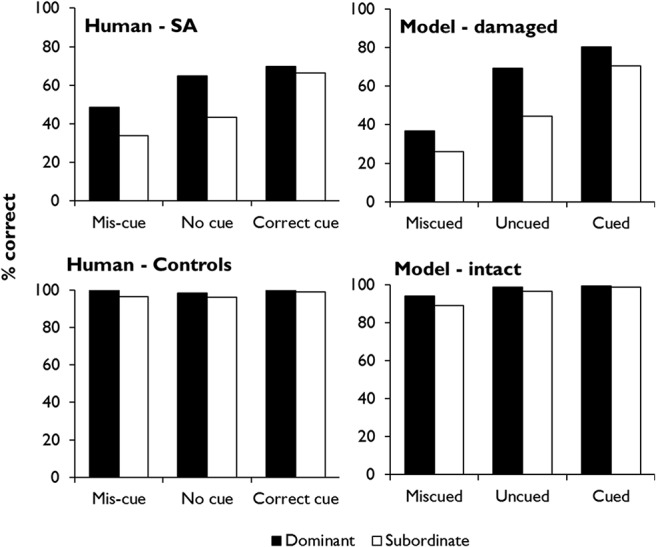
Target data and model performance for Simulation 1.

The top-left panel of Figure 9 shows performance on the task by seven SA patients studied by Noonan et al. (2010; see Simulation 1 for more further details). In the no-cue condition, patients were more successful when dominant, prepotent meanings were probed, relative to subordinate ones, and this result was attributed to impairment of controlled retrieval. Provision of correct contextual information improved performance for the subordinate meanings, so that these items reach a similar accuracy level to the dominant trials. This is thought to occur because the guiding context elicits strong, bottom-up activation of the trial-appropriate meaning, reducing the need for controlled retrieval. Incorrect contextual information, in contrast, had a negative effect.[Fig-anchor fig9]

To explore the effects of these manipulations in our model, we must first adopt a procedure by which the network can complete the task. We believe that responses in lexical association tasks of this kind are heavily influenced by the co-occurrence rates of the various response options in natural language contexts (see Barsalou et al., 2008). In the model, this information is represented by the activations of the prediction units. To simulate the task in the model, we therefore present a probe word as input, allow the model to settle and then read off the activations of the prediction units representing the four response options. The option with the highest activation is the one that the model considers most likely to co-occur with the probe and should be selected as the response.

Response selection is, however, a complex process. Human decision-making processes are typically stochastic in nature (e.g., Usher & McClelland, 2001) and, in the semantic domain in particular, regions of prefrontal cortex have been linked with resolving competition between possible responses (Badre et al., 2005; Thompson-Schill et al., 1997). To simulate the potential for error at the response selection stage, we add a small amount of noise, sampled from a Gaussian distribution, to each of the activations before selecting the option with the highest activation. The effect of this step varies according to the difference in activation between the most active option and its competitors. When the most active option far exceeds its competitors, the small perturbation of the activations has no effect on the outcome. But when two options have very similar activation levels, the addition of a small amount of noise can affect which is selected as the response. Therefore, this stochastic element introduces a degree of uncertainty about the correct response when two options appear similarly plausible to the model.

Finally, we also manipulate context as in the original experiment. On no-cue trials, the context units are assigned a random pattern of activity. On cued and miscued trials, the model processes a context word prior to the probe, which is consistent with either the trial-appropriate or inappropriate meaning of the word. For example, a trial where the model is required to match *bank* with *cashier* could be preceded by either *economics* or *plant*.

**Figure 10 fig10:**
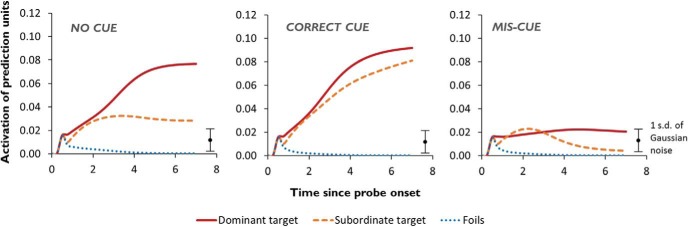
Activation of response options in the model with no control processes. The bars in the bottom right corner of each plot show the standard deviation of the Gaussian function used to add noise to each activation.

What happens when we use this procedure to test the model’s abilities using stimuli analogous to those shown in Figure 8? Figure 10 shows the mean activations of the prediction units representing the dominant and subordinate targets in this task, as well as the alternative options (these results are averaged across trials probing all 10 of the model’s homonyms; for further details, see Simulation 1). The results from the uncued condition illustrate the limitations of the model (which, at this stage, has no mechanism for controlled retrieval). As expected, the target relating to the dominant meaning is strongly activated such that, even when the stochastic response selection process is applied, the model is likely to distinguish the correct response from the foils. The subordinate target, however, is much less differentiated from the foils, so there is a greater chance that one of the three foils might be incorrectly selected. Context can modulate these effects in either direction. On correctly cued trials, the model’s expectations are shifted toward the trial-appropriate interpretation of the probe. Subordinate targets therefore become just as strongly predicted as dominant targets and are unlikely to be confused with foils. Conversely, when context primes the incorrect meaning of the word, the model fails to activate the target very strongly for either trial type. Thus, like the SA patients, the model’s ability to discriminate the target from its competitors is highly dependent on the degree to which the target receives strong bottom-up activation.[Fig-anchor fig10]

One of the reasons for this pattern of performance is that there are a range of activation states that the network can adopt for any given word, depending on the context in which it is processed. An appropriate context boosts the prediction signal for the subordinate targets because it constrains the model to reach an activation state for the probe that is consistent with its subordinate meaning. Our controlled retrieval process involves an *internally* generated source of constraint over the network that has a similar effect. Specifically, we force the model to process the probe *and* the various response options simultaneously. Our model has no prior experience of processing multiple words at the same time: During training, words are presented sequentially but not simultaneously. However, due to the graded, constraint-satisfaction properties of neural networks, at any given moment, the model attempts to settle into a state that is compatible with all of the inputs it is receiving. If, for example, the network is presented with both *bank* and *cashier* simultaneously, the hub units will settle into a hybrid state that is close to a viable representation of both words. As we show later on, this state is very different to that obtained if the network were presented with *bank* and *river*.

The effects of simultaneous processing of multiple inputs form the basis of the controlled retrieval process. We introduce an executive regulation mechanism that ensures the network’s activity is influenced by the response options as well as the probe during each trial. The goal of this mechanism is to ensure that the model settles into a state that is compatible with the probe and target but not with the other options. Of course, to begin with the model is not aware which of the four alternatives is the target. Controlled retrieval therefore takes the form of an iterative process that ensures that the model’s processing is initially influenced equally by all four alternatives, but as evidence accrues for one of the four options, this option is given greater influence over processing.

**Figure 11 fig11:**
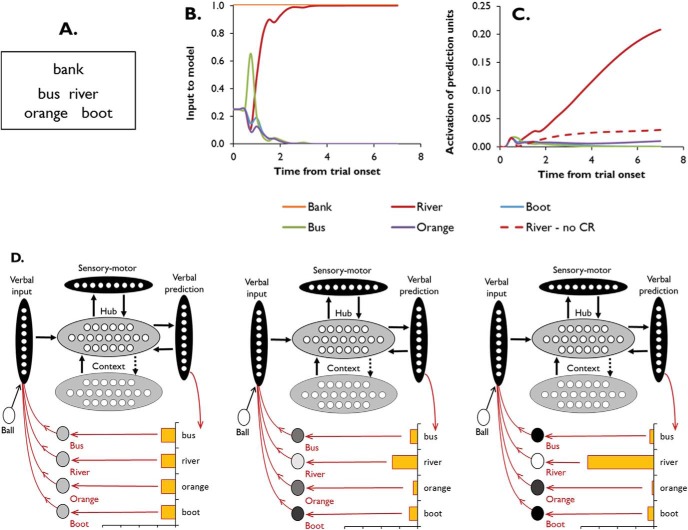
The controlled retrieval process. (A) The model is asked to decide which of four alternatives is most semantically related to *bank.* (B) Input to the model during settling. The model receives sustained input of the probe and a weighted combination of the possible responses. As the prediction for *river* strengthens, it comes to dominate the input. (C). Activation of prediction units during settling. The controlled retrieval process boosts the activation of *river*, relative to the level it would receive from processing of the probe alone (dashed line). (D) Graphical representation of settling. Elements of the controlled retrieval mechanism are shown in red.

An example of this process is shown in Figure 11, in which the model is required to select *river* as being linked to *bank* (see Panel A). Panel B shows the inputs to the model as it processes this trial and Panel C shows the activations of the prediction units for the four possible responses. Panel D provides a graphical illustration of the network at three points during processing. At any point in time, the input to the hub consists of *bank* and a weighted combination of the four response options. The weighting of the options, which is subject to top-down control, is determined by the values of their prediction units in the previous timepoint (for full implementation details, see Simulation 1). Before the network begins to settle, it considers each of the four options equally probable and they are all given equal weight as inputs to the model. This means that the model is constrained to settle into a state that is primarily influenced by *bank* but is also as compatible as possible with *bus, river, orange* and *boot*. Because there are states for *bank* that are rather close to *river* but no such states for the other options, the network begins to move toward an interpretation of *bank* that fits with *river*. As the network moves toward this state, the prediction value for *river* begins to increase while the values for the other options decrease. As a consequence, the control mechanism affords greater influence to this item, weighting it more heavily in the input to the hub. This in turn pushes the model further toward the *river-*compatible state, increasing its prediction value further. By the end of the trial, two things are apparent. First, the prediction value for *river* far outstrips that of the other options. This means that when the model comes to respond, it has no difficulty in identifying that *river* is the correct response. Note that this would not be the case without the application of the controlled retrieval process (see dashed line in Panel C). [Fig-anchor fig11]

**Figure 12 fig12:**
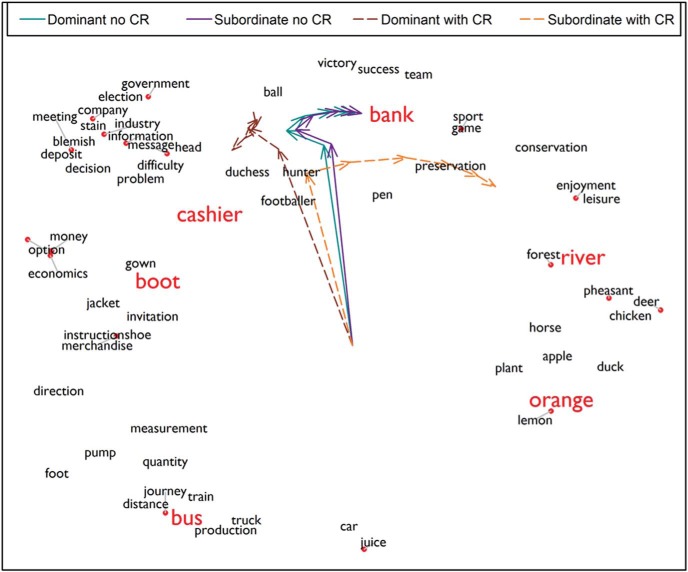
Model’s trajectory through semantic space during the *bank* trial. This plot illustrates the effect of controlled retrieval on the model’s internal representations. We first presented each word to the model in turn, allowed it to settle and recorded activity of the hub layer. Multidimensional scaling was used to plot the relationships between these states in a two-dimensional space (words used in the current trial are highlighted). We then recorded the activity of the hub units as the model completed the dominant and subordinate versions of the *bank* trial with and without controlled retrieval (CR). The lines plot the trajectory taken by the model through the semantic space as it settled. Without controlled retrieval, settling is determined solely by the identity of the probe, resulting in similar paths on dominant and subordinate trials, both of which end near the canonical representation of *bank*. Under controlled retrieval, settling is constrained by both probe and target. As a consequence, the model is deflected into areas of the semantic space somewhere between *bank* and either *cashier* or *tree.*

The second feature is that the input to the model is dominated by both *bank* and *river.* This means that the hub has been guided into an activation state that fits with both *bank* and *river*, rather than processing *bank* in its canonical sense. This is illustrated in Figure 12. As in previous figures, we used multidimensional scaling to plot the relationships between the model’s learned representations for all words. These relationships are averaged across many randomly generated contexts, so the representation of *bank* is closer to that of *cashier*, reflecting its dominant pattern of usage. In addition, we have plotted the states of the hub units when the model processes the *bank* trial, either in its subordinate sense (*bank-river*) or its dominant sense (*bank-cashier*). These activations were recorded at various points during processing, allowing us to plot the model’s trajectory through semantic space as it settles. Without controlled retrieval, the model processes *bank* in isolation and therefore settles into its most frequent pattern of usage, irrespective of whether the trial is probing the dominant or the subordinate meaning. In contrast, the controlled retrieval mechanism forces the behavior of the network to be influenced by the response options as well as the probe. This has the effect of shifting the network toward a different part of the semantic space, closer to the representation for either *cashier* or *river* (depending on which is available as a response). In a sense, therefore, the model ends the trial by “thinking about” a situation in which *bank* and the correct response could both appear.[Fig-anchor fig12]

### Summary

To use semantic information effectively, control processes are required to shape activation of knowledge to conform to the task at hand. One hypothesized control process is the notion of controlled retrieval, a top-down mechanism that guides activation of the appropriate semantic knowledge when the relevant representation is not automatically activated by bottom-up stimulus processing. We have implemented a mechanism for controlled retrieval in our model, which constrains the network to take multiple response alternatives into account when processing a particular word. Through an iterative feedback process, the network discovers which of the four options is most compatible with the probe and settles into an activation state compatible with that option.

Our model now has the key elements involved in semantic cognition: a set of semantic representations acquired through experience in the environment and a control process that regulates how these representations are activated and selected. We are now in a position to test how well the model’s behavior replicates human performance on semantic tasks. In the following section, we do this by probing (a) the ability of the intact model to perform semantic tasks in a similar way to healthy participants and (b) the ability of the model to mimic the effects of damage to either semantic representation or semantic control in patients with SD and SA, respectively. We test this in three simulations of verbal comprehension tasks.

### Simulation 1: Comprehension of Homonyms in Semantic Aphasia

In this simulation, we test the model’s ability to perform Noonan et al.’s (2010) homonym comprehension task, which we have already described. We also test the degree to which damage to the model’s control processes produces impairments similar to those observed in SA.

#### Target data

Noonan et al. (2010) tested seven patients with SA and eight age-matched healthy controls using the comprehension task described in the previous section (see Figure 8). The controls performed close to ceiling in all conditions but, as we have already described, the SA patients demonstrated (a) impairment on the task overall and (b) greater impairment for trials probing the subordinate meanings of words, and (c) a strong influence of context that interacted with meaning dominance. These data are shown on the left of Figure 9.

#### Test construction for simulation

To simulate Noonan et al.’s (2010) data, we constructed two trials for each of the 10 homonyms in the model’s vocabulary. Each trial consisted of a probe (the homonym), a target (semantically related to either its dominant or subordinate usage), and three unrelated foils. To assess the strength of the relationships between probes and targets, we computed the *co-occurrence rate* of each target given the probe. This value represents the proportion of times the probe was immediately followed by the target in the model’s training corpus. The co-occurrence rate for dominant targets (*M* = .077; range = .048–.099) was higher than that of subordinate targets (*M* = .033; range = .019–.052), indicating that the dominant targets were indeed more strongly associated with the probes in the model’s experience. In contrast, the foils always had a co-occurrence rate of 0 (i.e., they never occurred in the same context as the probe during training).

#### Simulation method

Testing of the model proceeded as follows. First, we instantiated the context in which the model would process the trial. When testing patients and controls, Noonan et al. (2010) presented a whole sentence that primed one particular interpretation of the probe. In the model, we presented a single word. On Correct Cue trials, we presented a word that was related to the trial-appropriate usage of the probe (e.g., on the *bank-cashier* trial, we presented *economics*). The network processed this word in the usual way. Having settled, the activation pattern over the hub units was copied to the context units, ready to influence processing when the network was presented with the probe and response options. The process was the same for miscue trials, except that the cue was related to the trial-inappropriate usage. On no cue trials, no cue word was presented; instead, we assigned the context units a random pattern of activity, so that no meaningful context was available to influence the decision-making process.

Next, we presented the probe and response options to the model for a total of seven time steps. At each point during processing, the input to the model consisted of the probe and a weighted combination of the four response options. To compute the weighting, the activation of the prediction units for the four options were subjected to a softmax transformation. The input value *I* for option *j* was given by the formula

$$I_{j}=\frac{exp\left(sP_{j}\right)}{\sum_{k=1}^{4}exp\left(sP_{k}\right)}$$

Where *P*_*j*_ denotes the activation of the prediction unit for option *j* and *s* is a constant that governs how sensitive the input values are to changes in the prediction values. The transformation ensured that the four inputs always summed to one but that options with larger prediction values were weighted more strongly. We set *s* to 200 in all simulations, based on pilot work.

At the end of processing, the prediction values for each of the four response options were recorded. Noise, sampled from a Gaussian distribution with a mean of 0 and standard deviation of 0.01, was added to each of them. Following this step, the option with the highest prediction value was taken as the model’s response.

This process was repeated 200 times for each trial (20 times in each of the 10 models trained with different starting weights) and the results averaged to provide a measure of neurologically intact performance in the model.

#### Damage

To simulate the performance of SA patients, we disrupted the executive mechanisms assumed to be impaired in this condition. First, we removed the controlled retrieval process. This meant that the model’s behavior was driven solely by the bottom-up activity elicited by the probe and the model was not constrained to find an activation state that fitted the response options as well. In addition, we increased the standard deviation of the Gaussian noise added at the response selection stage, from 0.01 to 0.045. This weakening of the selection process reflects the fact that SA patients also have difficulty with selecting among competing response options, which is assumed to be another important element of semantic control (Badre et al., 2005; Thompson-Schill et al., 1997). The figure of 0.045 was selected so that the model’s overall accuracy on the task was as close as possible to the patients’.

#### Results

Model performance is presented in Figure 9, alongside the results reported by Noonan et al. (2010). Noonan et al. (2010) analyzed their human data using a 2 (impairment) × 2 (dominance) × 3 (cue) ANOVA. We performed the same analyses on the model data, treating each of the 10 trained models as a separate case in the analysis (see Table 1). In the human data, SA patients showed larger effects of the dominance manipulation than controls, demonstrating particularly poor comprehension of subordinate meanings. They also showed larger effects of the cue manipulation and there was a three-way interaction between these factors, indicating that the advantage for dominant meanings was attenuated when a correct cue was provided. All of these effects were replicated in the model.[Table-anchor tbl1]

#### Effects of alternative forms of damage

**Figure 13 fig13:**
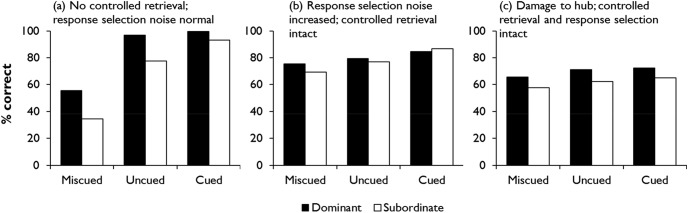
Model performance in Simulation 1 under alternative forms of damage.

The performance of the model under damage (no controlled retrieval plus additional noise at the response selection stage) closely resembles the pattern shown by patients with SA. It is important to establish the degree to which these effects are a consequence of the specific type of damage we applied and not simply a more general consequence of weakening the model. To assess this, we tested the model under three different types of damage (see Figure 13). Panel (a) shows the effect of removing the model’s ability to perform controlled retrieval without changing the noisiness of response selection. Under these conditions, the model demonstrates a strong cueing effect, indicating that controlled retrieval is important for supporting performance when contextual information is absent or misleading. However, overall levels of performance were higher than observed in SA patients, suggesting that these patients’ control deficits extend beyond a difficulty with controlled retrieval. When we disrupted response selection by increasing the level of noise, but allowed the model to use controlled retrieval (Panel b), there was a general depression in performance but little effect of meaning dominance or cueing. Finally, we tested the effect of removing connections projecting in and out of the model’s hub layer (Panel c). This form of damage (which we will use to simulate SD patients in later simulations) degrades the model’s representational substrate but not its control processes. Again, this form of damage degraded performance but did not produce the strong effects of cue type observed in SA.[Fig-anchor fig13]

#### Accounting for the success of controlled retrieval

**Figure 14 fig14:**
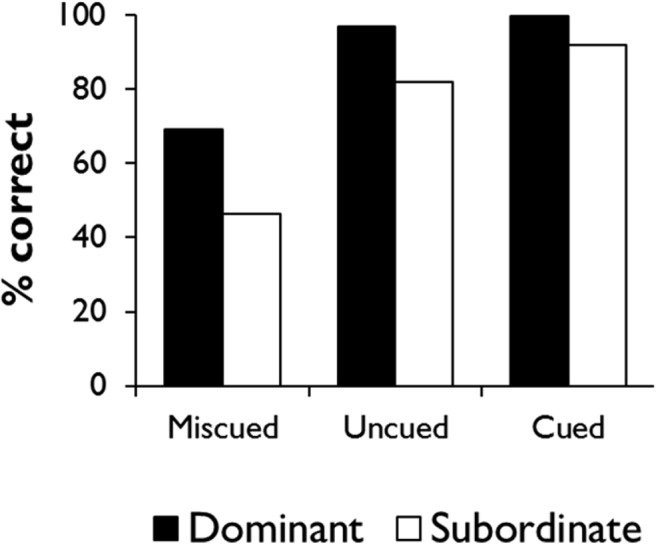
Performance of the intact model in Simulation 1 with an alternative form of controlled retrieval.

There are two potentially important elements of the controlled retrieval mechanism that may explain its success in identifying the homonyms’ nondominant associations. First, it forces the network’s processing of the probe to be colored by the available potential responses and second, it controls the relative weighting of those response options based on the network’s current predictions. Is the adaptive weighting of the response options necessary, or would it be sufficient to simply provide all of the alternatives as input with equal influence afforded to each? To investigate this, we tested an alternative form of the controlled retrieval mechanism in which the response options were included in the input to the hub but they were not weighted based on feedback from the prediction units. Instead, each response option received a static weighting of 0.25 (so that the four response weights still summed to one). Performance of this version of the model is shown in Figure 14. It performed well in the cued condition and for the dominant meanings in the uncued condition, but it was much less successful in the other conditions (cf. the intact model in Figure 12). This indicates that an iterative control process, whereby feedback is used to continually adjust the degree to which each option influences the network’s state, is critical in ensuring that the network discovers the correct activation state for weak or nondominant semantic relationships.[Fig-anchor fig14]

#### Discussion

The intact model, with its controlled retrieval mechanism, was able to select which of four words was associated with a presented homonym, even when the target related to its subordinate meaning. When the controlled retrieval process was removed and the model’s response selection process impaired, the results closely resembled performance that of patients with SA. Performance on subordinate trials was disproportionately affected and the model became much more reliant on context for guiding it toward the correct response. These results indicate that the controlled retrieval process we have implemented provides a plausible account of how top-down control influences performance on this task.

### Simulation 2: Effects of Frequency, Imageability, and Semantic Diversity on Semantic Judgments in SD and SA

We have demonstrated that damage to the model’s controlled retrieval mechanism produces deficits in a verbal comprehension task similar to those observed in SA patients with semantic control deficits. In the second simulation, we tested the model’s ability to mimic the *divergent* patterns of impairment exhibited by SD and SA patients. As we have already alluded to, even when patients with SD and SA show similar levels of impairment on semantic tasks, the details of their impairments are very different. These qualitative differences are thought to indicate damage to semantic representations or to semantic control processes respectively (Jefferies, 2013; Jefferies & Lambon Ralph, 2006; Rogers et al., 2015). If our model provides an accurate account of both semantic representation and control, then damage to these two elements of the model should simulate the divergent effects observed in these two disorders. To test this, we focused on data reported by Hoffman, Rogers, and Lambon Ralph (2011b), in which matched groups of SD and SA patients completed a verbal semantic judgment task. Hoffman et al. investigated the psycholinguistic factors influencing performance in each group. Despite similar overall levels of impairment, the two groups displayed divergent effects of word frequency, imageability, and semantic diversity, which were hypothesized to be a consequence of impaired semantic representation versus control. Here, we tested whether the model would display similar effects under damage to either its representational hub or its control processes.

#### Target data

Hoffman et al. (2011b) presented data from 13 patients with SA and 13 with SD. Patients completed a semantic judgment task in which they were presented with a probe word and asked which of three alternatives was similar in meaning (Jefferies et al., 2009). A multiple regression approach was used to investigate the factors that governed each group’s performance on the task. Specifically, we investigated how the following three psycholinguistic factors influenced the patients’ ability to make semantic judgments.

#### Semantic diversity

This is a measure of the contextual variability of words, derived empirically by determining the level of similarity among all the contexts in which a particular word is used (Hoffman et al., 2013b). The measure is motivated by the idea, implicit in distributional approaches to semantics, that the meaning of a word changes every time it is used in a different context. On this view, *all* words are somewhat polysemous, with the degree of variation in their meaning depending on the degree to which they are used in a wide variety of contexts (Cruse, 1986; Hoffman et al., 2013b; Landauer, 2001). The semantic diversity measure assesses this variation empirically, through analysis of a large corpus of text samples. Words with low semantic diversity are used in a restricted set of closely related contexts, while those with high diversity are found in a wide range of disparate contexts.

SA patients showed a strong negative effect of semantic diversity, performing more poorly with words that are used in a wide range of different contexts. We hypothesized that this is because the meanings of highly diverse words change when they are used in different situations. As consequence, activating the task-appropriate semantic representation for such words places greater demands on controlled retrieval processes (just as these processes were necessary for activating the appropriate representation for homonyms in Simulation 1). In contrast, performance in the SD group was not affected by semantic diversity, in line with the idea that these patients’ deficits are not linked with executive impairment.

#### Imageability

Imageability refers to the ease with which a word elicits mental imagery, and is therefore an index of how concrete or abstract a word is (Paivio, Yuille, & Madigan, 1968). Both patient groups displayed better comprehension of highly imageable words. In the case of SD, we have hypothesized that, because they lack grounding in the S-M experience, abstract concepts are represented weaker in the semantic hub. As a consequence, damage to the hub has a particularly adverse effect on these words (Hoffman, 2016; Hoffman & Lambon Ralph, 2011). The explanation for SA patients is less clear. Abstract words tend to be more semantically diverse than concrete words (Hoffman et al., 2013b); however, this is not a complete explanation as the imageability effect remained significant in a simultaneous regression that controlled for semantic diversity.

#### Frequency

Word frequency has an almost ubiquitous effect in language processing tasks. In SD, we observed a strong frequency effect, with much better comprehension of more frequent words. This effect has been observed in many studies of SD (Bozeat et al., 2000; Funnell, 1995; Jefferies et al., 2009) and reflects the tendency for concepts that are encountered more frequently to be represented more robustly in the semantic system (Rogers & McClelland, 2004). In contrast, frequency effects are typically weak or absent in patients with SA (Almaghyuli, Thompson, Lambon Ralph, & Jefferies, 2012; Hoffman, Jefferies, & Lambon Ralph, 2011a; Jefferies & Lambon Ralph, 2006). Hoffman et al. (2011b) demonstrated that this was because high frequency words tend to be highly semantically diverse and therefore place high demands on the patients’ impaired control processes, counteracting the usual advantage for these words. When semantic diversity was controlled for statistically, a small effect of frequency did emerge for SA patients, although this was much weaker than the effect observed in SD.

In summary, SA patients with damage to control processes displayed poor comprehension of words of high semantic diversity, while representational damage in SD was characterized by especially poor comprehension of low frequency words. Both groups were better at making judgments to more imageable words. We investigated whether the model would display similar behavior under damage intended to mimic each disorder.

#### Test construction for simulation

In order to investigate the influence of psycholinguistic properties on the model, it was vital that our training corpus embodied these properties in as realistic a fashion as possible. We therefore used analyses of actual language use to guide construction of the training and testing environments. We begin by describing how each of the psycholinguistic variables were operationalized in the training environment.

#### Semantic diversity

The model’s training corpus was generated by sampling from a set of topics, each of which consisted of a probability distribution over a subset of the words known to the model. All the words in the model’s vocabulary appeared in at least three different topics, but the topics were designed such that some words appeared in a restricted set of topics while others could occur in many disparate topics. To quantify this variation, we computed a semantic diversity value for each word. Semantic diversity is calculated by performing latent semantic analysis on a large corpus of natural language samples (Hoffman et al., 2013b). The result is that each sample (or context) in the corpus is represented by a vector that describes its location in a high-dimensional semantic space. Contexts that contain similar words have similar vectors and, under the distributional principle, are assumed to be similar in their semantic content. To compute the semantic diversity for a particular word, one calculates the pairwise similarities between the vectors representing all of the contexts that contain the word. This value is then log-transformed and its sign reversed, so that higher values indicate greater dissimilarity between the various contexts in which the word is used.

The exact same process was performed on the model’s training corpus to compute semantic diversity values for each of the words in its vocabulary. The least diverse word (*deer*) had a value of 0.13 and the most diverse (*lorry*) 0.76. In previous work (Hoffman et al., 2013b; Hoffman & Woollams, 2015), we have proposed that the semantic representations of highly diverse words are very variable and that this makes them more difficult to process in semantic tasks. To test whether this held true in the model, we presented each word in 64 different contexts (i.e., in the context of each word in its vocabulary) and recorded the representations over the hub units. We then computed the pairwise similarities between the representations for the same word in these different contexts, providing a measure of the word’s *representational consistency*. There was a strong negative correlation between consistency in representation and semantic diversity, *r* = −0.36, *p* = .004. Thus, as predicted, the emergent consequence of words being used in a broad range of contexts is that they develop semantic representations that vary greatly across contexts.

#### Imageability

In their analyses of patient data, Hoffman et al. (2011b) treated imageability as a continuous variable. In the model, however, imageability is implemented as a binary distinction (the model is trained to associate the 22 concrete words with S-M properties, while no such training is provided for the 32 abstract words). Nevertheless, we were keen to ensure that the relationship between imageability and semantic diversity in the model accurately reflected that seen in real language. Because the verbal input units in the model notionally represent real English words, we obtained the semantic diversity of those words in a published database derived from the British National Corpus (Hoffman et al., 2013b). We found that the concrete words had lower semantic diversity values (*M* = 1.50) than the abstract words (*M* = 1.80; *t*(52) = 5.28, *p* < .001). This relationship is also present in larger samples of words (Hoffman et al., 2013b). We therefore ensured that, in the model, the concrete words had lower semantic diversity values than the abstract words (concrete *M* = 0.49; abstract *M* = 0.56).

#### Frequency

Word frequency was manipulated in the model by varying the number of topics particular words appeared in, the probability of selection within those topics, and by ensuring that some topics were sampled more often than others. As a consequence, the most frequent word (*lorry*) occurred in the training set 17 times more often than the least frequent word (*team*). To ensure that the relationships between frequency and the other psycholinguistic variables accurately mimicked those seen in natural language, we again investigated the properties of the real English words upon which the model’s vocabulary was based. We found a strong positive correlation between frequency and semantic diversity, *r* = .57, *p* < .001: higher frequency words tended to be more semantically diverse. We therefore replicated this effect in the model, *r* = .64, *p* < .001. Because frequency and imageability are both correlated with semantic diversity, to investigate the relationship between these two variables, we computed their partial correlation while controlling for semantic diversity. There was no relationship between frequency and imageability, *r* = −0.08, *p* = .53. Accordingly, we ensured that no such relationship was present in the model’s training environment, *r* = .15, *p* = .28.

#### Test construction

To test the model, a semantic judgment task was constructed that corresponded as closely as possible to the test used by Hoffman et al. (2011b) to investigate performance in SD and SA patients. Each trial in the neuropsychological study comprised a probe, a target that was similar to it in meaning and two unrelated foils. We constructed one such trial for each of the 22 concrete and 32 abstract words in the model’s vocabulary. Probe-target pairings for abstract words are shown in Figure 2. The targets on concrete trials were always a concrete item from the same category as the probe.

In addition to ensuring that the distribution of psycholinguistic properties in the model was closely representative of real language, it was critical that the materials used to test the model closely matched the test used with the patients. For this reason, when we constructed the semantic judgment test for the model, we paid close attention to the relationship between the probe and target on each trial. Performance in the model is strongly influenced by the co-occurrence rate of the target with the probe during training. When the target frequently occurs immediately after the probe during training, the model learns to strongly activate the target’s prediction unit when it is presented with the probe. This strong prediction value makes it easy for the model to select the target as the correct response. Because co-occurrence rate is an important driving factor in the model, we investigated how this property is related to other psycholinguistic variables in real language. We did this by taking each trial from the neuropsychological test used by Hoffman et al. (2011b) and finding each occurrence of the probe in the British National Corpus (British National Corpus Consortium, 2007). We then computed in what proportion of those occurrences, the target appeared in the next 10 words in the corpus. This represented the co-occurrence rate of the target with the probe in a large corpus of natural language.

We used a simultaneous multiple regression model to investigate how frequency, imageability and semantic diversity were related to co-occurrence rates in the neuropsychological test. Trials featuring higher frequency words and lower semantic diversity words tended to have higher co-occurrence rates (see Table 2). When constructing the test for the model, we were careful to replicate this pattern (see lower half of Table 2). As a result, the psycholinguistic properties we investigated in the model were related to the difficulty of individual trials in ways that accurately reflect the neuropsychological test used to collect the patient data.[Table-anchor tbl2]

#### Simulation method

The procedure for testing the model was similar to Simulation 1. No contextual information was available to patients in the Hoffman et al. (2011b) study, so the context layer was reset to a random pattern of activity at the start of each trial. The model was then presented with the probe. The predictions for the three response options were processed by the controlled retrieval mechanism, which iteratively regulated the network’s activity as described previously. At the end of the processing window, the most active option, after the addition of Gaussian noise, was selected as the response. Each trial was presented 200 times and the results averaged to give a measure of intact model performance.

#### Damage

To simulate semantic control deficits in SA, we again removed the controlled retrieval mechanism and increased the standard deviation of the Gaussian noise added at the response selection stage. This was increased from 0.01 to 0.04; this value was selected because it gave overall accuracy levels that were very closely matched to the target dataset. Again, each trial was presented 200 times and the results averaged. To simulate damage to the semantic hub in SD, we removed a certain proportion of the links projecting in and out of the hub layer, thus degrading the function of this crucial element of the model (Rogers et al., 2004). We removed 30% of the links as this level of damage gave the closest fit to the target dataset in terms of overall accuracy. Each of the 10 trained models was damaged 20 times and tested, again yielding 200 presentations for each trial.

#### Results

**Figure 15 fig15:**
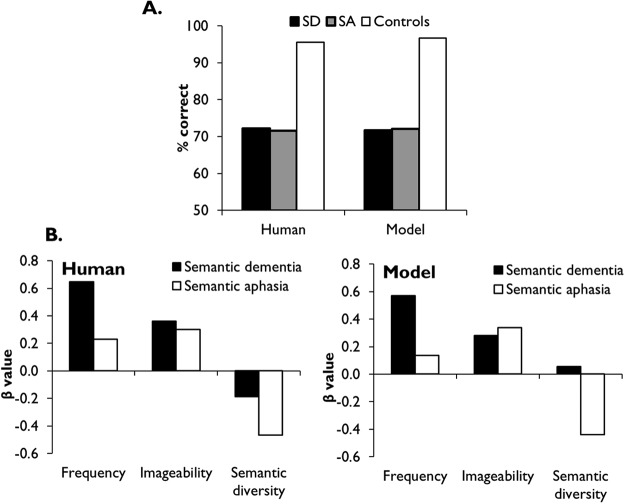
Target data and model performance for Simulation 2. (A) Accuracy levels for human data and in the model (healthy control data taken from Hoffman et al., 2013b; patient data from Hoffman et al., 2011b). (B) Beta values from linear regression models that used psycholinguistic properties to predict human and model performance on individual trials.

Accuracy in the model and in the target dataset are presented at the top of Figure 15. Without damage, the model completed 97% of trials accurately, which is similar to the level achieved by healthy participants completing the neuropsychological test. Under damage, accuracy levels in the model were closely matched to the patients. To investigate the influence of psycholinguistic properties on model performance, we performed a linear regression analysis in which probe frequency, imageability and semantic diversity were used as predictors of performance on individual trials. The results are shown in Table 4, alongside the corresponding results from the patient data. The correlation matrices for both analyses are shown in Table 3 and the beta weights are illustrated graphically in Figure 15. Results in the model show strong convergence with those in the target dataset. Imageability had a positive effect on model performance under damage to both control processes and representations. Similarly, both SD and SA showed a positive effect of imageability. Following damage to control processes, the model showed a weak positive effect of frequency and a strong negative influence of semantic diversity. This was precisely the pattern observed in SA patients. In contrast, damage to the model’s hub layer resulted in a strong positive effect of frequency and no effect of semantic diversity. Likewise, SD patients showed a strong frequency effect and a nonsignificant semantic diversity effect.[Fig-anchor fig15][Table-anchor tbl4][Table-anchor tbl3]

To determine the degree to which probe-target co-occurrence rates were responsible for these results, we added this factor as an additional predictor to the regression analyses. Inclusion of co-occurrence rates did not improve ability to predict performance in the model with damage to the hub, Δ*R*^2^ = 0.011; *F*(1, 49) = 0.93, *p* = .34. This indicates the co-occurrence rates of the probes and targets were not a major factor in determining how the model performed following representational damage. In contrast, there was a significant improvement in the fit for the model with damage to control processes, Δ*R*^2^ = 0.291; *F*(1, 49) = 35.7, *p* < .001. Following the addition of co-occurrence rates, imageability remained a significant predictor of performance (β = 0.28, *p* = .005) but frequency was not (β = −0.04, *p* = .73) and nor was semantic diversity (β = −0.17, *p* = .19). This indicates that, when the model’s control processes are damaged, the rate with which the target and probe have occurred together in its prior experience is the main determinant of whether it is able to match them at test. The effects of frequency and semantic diversity in this case can be attributed to this underlying factor.

Finally, to test for potential interactions between imageability, frequency, and semantic diversity we ran linear mixed effects models on the results (with model number and probe as random factors). There were no interactions following damage to the network’s control processes. However, there was an interaction between frequency and imageability for the models with damaged hub representations (χ^2^ = 2.58, *p* = .01). Under this type of damage, the effect of frequency was larger for the concrete words.

#### Discussion

We investigated the model’s ability to make semantic judgments following damage to either its control processes or its representational substrate. Although the overall level of impairment in these two cases was matched, the factors underpinning the deficits were different and closely matched the divergent patterns observed in patients with SD versus SA. When the model’s control processes were disrupted, it became highly sensitive to the semantic diversity of the words being probed, performing poorly with words that appeared in many different contexts. It also displayed a modest sensitivity to word frequency. Importantly, the rate at which the probe and target had co-occurred during training appeared to be the root cause of these effects. When a word appears in many different contexts, it shares semantic relatedness with a wide range of different words but co-occurs with each of those other words less frequently. As a consequence, when the model is presented with such a word as a probe, it activates weak predictions for a wide range of words, each of which could potentially occur in the same context as the probe. These weak predictions make it hard to differentiate the target from the other response options. In the intact model, the controlled retrieval process ameliorates this problem by forcing the network into a state in which it *does* have a strong expectation of the target appearing, as seen in Simulation 1. But when this process does not function, the weak prediction for the target does not strongly differentiate it from its foils. Disruption to the response selection process, by making this stage nosier, further exacerbates the problem. In short, the model suggests that SA patients find it hard to make semantic decisions about words with high semantic diversity because these words weakly activate a wide range of potentially associated words. Reliably identifying the correct word in these circumstances requires top-down support from the executive system, which is not available to these individuals. The effects of homonym comprehension presented in Simulation 1 can be considered a special case of this more general effect of contextual variability.

In contrast, when the model’s semantic hub was damaged, performance was not governed by semantic diversity; instead, the model demonstrated much better comprehension of higher frequency words. This replicates effects seen in patients with SD across a range of semantic tasks (Bozeat et al., 2000; Funnell, 1995; Jefferies et al., 2009). The robust semantic representations of high frequency words were explored by Rogers and McClelland (2004). They demonstrated that when the model encounters a word frequently, it has many opportunities to learn the appropriate patterns of activation for this word. As a consequence, it develops a robust representation of the word early in the learning process. When the representational system is later degraded by damage, the strong representations of high frequency words are affected to a lesser extent. Semantic diversity has little effect on performance in the hub-damaged model because the controlled retrieval process is unaffected. The model can therefore compensate for the weak target activation on high semantic diversity trials, provided that it still has a reasonably intact representation for the probe to begin with. These results are consistent with the widely held view that SD is a relatively pure disorder of semantic representation and that executive control processes function well in this condition (Jefferies & Lambon Ralph, 2006).

Under both types of damage, the model displayed better comprehension of concrete relative to abstract words. This effect cannot be attributed to differences in frequency, semantic diversity or co-occurrence rates, which were all controlled for in the analyses. The key difference must therefore be the association of concrete words with S-M properties. We have suggested previously that this results in concrete words developing richer semantic representations, explaining the more preserved comprehension of such concepts in most SD patients (Hoffman, 2016; Hoffman & Lambon Ralph, 2011). The meanings of concrete words also tend to be acquired earlier in life than those of abstract words (Stadthagen-Gonzalez & Davis, 2006). This is true in the model. Although the model is exposed to both concrete and abstract words from “birth,” it develops representations for concrete words more quickly because they are mapped consistently with their S-M properties. This early acquisition also ensures that concrete words have robust representations in the face of damage (Ellis & Lambon Ralph, 2000), just as high frequency words do.

Another factor of potential importance is the presence of feedback connections from the model’s S-M units to the hub. As the model begins to process a concrete word, it quickly activates a strong, contextually invariant pattern of S-M activity. This emerging S-M representation feeds back into the hub layer, providing an important additional source of constraint for the hub as it settles into a coherent representation of the word (note that while abstract words do come to activate some S-M information through acquired embodiment, they do so in a weaker and more contextually varying fashion). This feedback activation provides additional support for the hub representations of concrete words, which partially ameliorates damage to this element of the network. Feedback from S-M units to the hub may also have a beneficial effect when control processes are impaired because it ensures that the network settles into a state consistent with the S-M properties activated, and the probe and target share some S-M properties.

### Simulation 3: Taxonomic and Associative Relationships in SD and SA

In Simulation 2, we explored how the model made semantic judgments to concrete and abstract words under damage. In the final simulation, we restricted our attention to concrete words and considered how the model performed when different types of semantic relationship were probed. There has long been an important distinction made between taxonomic semantic relationships, between items which share S-M properties, and associative relationships, between items which share few properties but which co-occur in particular spatiotemporal contexts (Alario et al., 2000; Lin & Murphy, 2001; Perea & Gotor, 1997; Seidenberg et al., 1984). As our model codes semantic structure based on the integration of S-M and contextual information, it should be sensitive to both types of relationship. We have already demonstrated that our model’s unitary semantic space codes information about both types of relationship. Here, we investigated the model’s ability to make semantic judgments on the basis of association as well as S-M similarity, when intact and under damage. Patients with SD show similar levels of impairment when asked to match items either on the basis of taxonomic similarity or association (Hoffman et al., 2013a). SA patients also show similar levels of impairment for both types of relationship (Jefferies & Lambon Ralph, 2006; Noonan et al., 2010). We tested the model’s ability to simulate these patterns.

#### Target data

**Figure 16 fig16:**
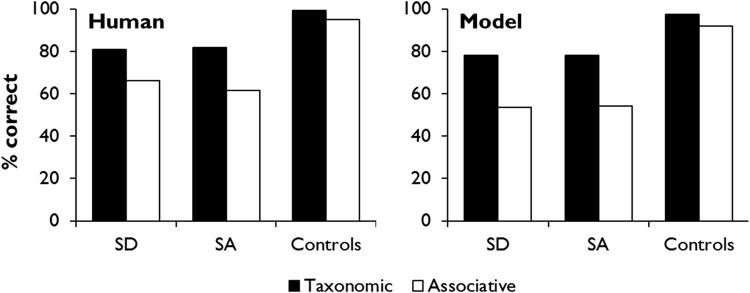
Target data and model performance for Simulation 3.

Data for taxonomic and associative semantic decisions are taken from Hoffman et al. (2011b). For taxonomic decisions, we took the most highly imageable trials from the semantic judgment task described in Simulation 2. For associative decisions, we investigated performance on the word version of the Camel and Cactus Test (Bozeat et al., 2000). In this test, patients were presented with the name of a concrete probe and asked to decide which of four items was semantically related to it (e.g., does *camel* go with *cactus, rose, sunflower,* or *tree*?). The target always belonged to a different category to the probe. It is important to note that the two tasks were designed independently and are not matched on all factors that might influence performance (e.g., word frequency, number of alternatives). Nevertheless, they provide a benchmark for assessing relative levels of performance across patient groups. Both SA and SD patients performed poorly on both tasks (see Figure 16). Healthy control data for the Camel and Cactus Test was reported by Bozeat, Lambon Ralph, Patterson, Garrard, and Hodges (2000) and for the semantic judgment task by Hoffman et al. (2013b). These are also displayed in Figure 16.[Fig-anchor fig16]

#### Test construction for simulation

For the taxonomic decisions, we used the 22 concrete word trials from Simulation 2. For the associative decisions, we constructed 22 new trials, each with a target and three unrelated foils. The target had frequently occurred alongside the probe during training (mean associative strength = 0.039) but belonged to a different category and consequently had different S-M properties. Foils also belonged to different categories to the probe.

### Simulation Method: Procedures for Damaging and Testing the Model Were Identical to Simulation 2

#### Results

The undamaged model was able to make both taxonomic and associative decisions at over 90% accuracy (see Figure 16). This is comparable with performance in healthy individuals. Patients with SA and SD were impaired for taxonomic and associative judgments to a similar extent. In all groups, performance was slightly worse for associative judgments, which may reflect the fact that these judgments required selection from four alternatives, rather than three. In any case, the model demonstrated a similar pattern of behavior: Both types of damage had a similar effect on both taxonomic and associative judgments, with poorer performance on the whole for the associative decisions.

#### Discussion

In addition to matching concrete items that belonged to the same semantic category, the model was able to match associated items that shared no S-M properties. This indicates that the network’s single set of semantic representations simultaneously coded information about category structure, based on shared S-M features and concept co-occurrence, as well as associative relationships based on concept co-occurrence alone. Both types of judgments were impaired to a similar extent under damage to either the hub representations or control processes, mirroring results from SD and SA patients.

## General Discussion

We have presented a connectionist model of semantic cognition that represents a theoretical advance on several fronts. The starting point for our model is the established view that semantic representation arises from the convergence of multiple, modality-specific sources of information on a central semantic “hub” (Lambon Ralph et al., 2010, 2017; Patterson et al., 2007; Rogers et al., 2004). In learning to map between the names of objects and their sensory-motor (S-M) properties, the hub develops conceptual representations which capture the underlying similarity structure among the objects. We have significantly extended the theoretical reach of this framework by allying it with the distributional principle: the idea that semantic relationships can also be inferred from the co-occurrence of words or objects in the same contexts (Firth, 1957; Griffiths et al., 2007; Jones & Mewhort, 2007; Landauer & Dumais, 1997; Lund & Burgess, 1996; Sadeghi et al., 2015). Our model was presented with sequences of concepts and was required to predict which concepts are likely to co-occur with one another, by making use of a recurrent architecture that buffers recent experience (Elman, 1990). Under these twin pressures—to map between words and S-M experiences and to predict which words co-occur with one another—the system developed semantic representations that coded the relationships between concepts based on a fusion of S-M similarity and concept co-occurrence. This proved to have a number of advantages:

1 As in previous connectionist approaches to semantic representation (e.g., Rogers et al., 2004), the model represents items with similar S-M properties as semantically related to one another. In addition, by learning about the contextual co-occurrence of items, the network also becomes sensitive to associative relationships between objects that have entirely distinct S-M properties.
2 Because of its adherence to the distributional principle, the model is able to learn about abstract concepts, which have few direct links to S-M experiences and have until now been largely overlooked in computational models of semantic cognition. Although the model is never explicitly trained to associate abstract words with S-M experiences, it does come to link S-M information with abstract words indirectly, by virtue of their association with concrete items. The model therefore provides a mechanism by which the meanings of abstract concepts can become partially grounded in the physical world. This addresses a fundamental criticism that has often been levelled at approaches based on the distributional principle: that they lack grounding in S-M experience (Glenberg & Robertson, 2000).
3 The model’s representations are context-sensitive, allowing for the multiple meanings of homonyms to be represented distinctly and, perhaps more significantly, for the representations of all words to vary in a graded fashion according to the particular context in which they are being used. This is made possible by the model’s recurrent architecture, whereby network activity at any point in time is influenced jointly by the identity of incoming stimulus from the environment *and* by the network’s buffered copy of its own internal state following processing of the previous stimulus.

In addition to these advances regarding the nature of semantic representation, the model breaks new ground by incorporating a mechanism for executive regulation of activity in the semantic system. Control processes are known to play an important role in semantic cognition, by providing top-down influences which ensure that the activation of semantic information is appropriately tailored to the current goal or context (Badre & Wagner, 2002; Jefferies, 2013; Jefferies & Lambon Ralph, 2006; Thompson-Schill et al., 1997). One such hypothesized control process is a “controlled retrieval” mechanism that is thought to direct semantic activation when automatic processing of the stimulus fails to generate a suitable response. Earlier, we gave the example of the concept of a *bee*, which might automatically bring to mind their most common properties such as buzzing, flying, making honey, and living in a hive. When completing a semantic task that requires one to match *bee* with *pollen*, however, one has to go beyond these dominant associations and focus on a specific context in which bees act as pollinators of flowers.

For the first time, we have proposed and implemented a computational mechanism for performing controlled retrieval. We tested this mechanism using a standard semantic task, in which participants are asked to decide which of a number of words is related in meaning to a probe word. The controlled retrieval mechanism ensures that the network’s activity is influenced by the word whose meaning is being probed but also, simultaneously, by the possible responses available. The network is constrained to find an activation state that is consistent with both the probe word and with one of the available responses. Through an iterative feedback process, the network is able to discover which of the response options is most compatible with the probe. In effect, the model ends up “thinking about” the probe in a way that is compatible with one of the available options.

In Simulation 1, we tested this mechanism by probing the model’s ability to select words related to the dominant and subordinate meanings of homonyms. We found that the model could successfully complete the task, but that damage to the controlled retrieval process resulted in deficits that mimicked those of patients with SA, who have impaired semantic control processes. In Simulation 2, we investigated the differential effects of damaging either the control processes or the model’s representational system, again in a verbal comprehension task. Damage to these two elements produced qualitatively different patterns of impairment, with respect to the effects of frequency, imageability, and semantic diversity. These divergent profiles closely matched the effects seen in patients with SA and SD, indicating that the model’s performance under damage is consistent with the hypothesized causes of semantic impairment in these two disorders. Finally, in Simulation 3 we found that damage to either control processes or representations had similar effects on judgments of taxonomic and associative semantic relationships, again mirroring results in patients with SD and SA.

In this discussion, we will consider the contribution of our model in developing a full neurocognitive theory of semantic cognition. We will also note some areas that the model does not address at present and consider how these might be addressed in the future.

### The Neural Basis of Semantic Cognition

There is now a large body of data concerning the network of brain regions involved in semantic cognition (see, e.g., Binder, Desai, Graves, & Conant, 2009). In this section, we consider how our model fits with current perspectives on the organization of the semantic neural network and note where it makes explicit predictions about the function of this network.

Our model uses a hub-and-spoke architecture (Lambon Ralph et al., 2010; 2017; Patterson et al., 2007; Rogers et al., 2004), which proposes that a distributed network of specialized regions (termed spokes) represents properties in particular sensory, motor and linguistic modalities, while the hub develops pan-modal, generalizable conceptual representations by virtue of its intermediary role (for related views, see Damasio, 1989; Garagnani & Pulvermüller, 2016; Simmons & Barsalou, 2003). As our focus was on central semantic representation, we did not attempt to represent the spoke regions in any detail in the model. We represented S-M properties using simple patterns over a single set of units; but in practice we believe that this information is coded across a range of specialized sites (see Binder & Desai, 2011; Rice, Hoffman, & Lambon Ralph, 2015). Verbal information was represented by verbal input units and prediction units, which we propose are supported by perisylvian language regions in the superior temporal cortex. We have not attempted to specify the function of these regions in any detail; undoubtedly there is a great deal of acoustic and phonological processing that is beyond the scope of our model. Other recent connectionist models have, however, sought to specify the functions of various spoke regions in a neuroanatomically constrained fashion (Chen, Lambon Ralph, & Rogers, 2017; Ueno, Saito, Rogers, & Lambon Ralph, 2011).

In the present work, we have focused on the structure and function of the central hub. In recent years, the ATL has emerged as the most likely neuroanatomical region underpinning this function. Converging evidence for the importance of this region comes from studies observing functional activation using PET and fMRI (Humphreys et al., 2015; Spitsyna, Warren, Scott, Turkheimer, & Wise, 2006; Vandenberghe, Nobre, & Price, 2002), damage to this area in SD patients (Butler et al., 2009; Mion et al., 2010), transcranial magnetic stimulation (Pobric et al., 2007), MEG (Marinkovic et al., 2003) and intracranial electrode recording (Nobre, Allison, & McCarthy, 1994; Shimotake et al., 2015). In all cases, the ATL, and in particular its ventral surface, has been associated with the representation of multimodal semantic knowledge, in line with the proposed hub function (Lambon Ralph et al., 2017). On this view, damage to this central, pan-modal element of the semantic system gives rise to the severe, multimodal semantic deficits observed in SD patients (Rogers et al., 2004). To simulate SD in our model, like Rogers et al. (2004), we damaged the hub units. We found that the model’s verbal comprehension performance under these conditions closely mimicked the pattern seen in SD. This supports the view that the ATL functions as an integrative representational hub, developing conceptual representations based on inputs from multiple verbal and nonverbal modalities.

The ventral parietal cortex (VPC) is also frequently implicated in semantic cognition though its function is less clear. Some authors have suggested that it plays a representational role similar to that of ATL. Specifically, it is claimed that semantic representation requires two distinct hubs (Binder & Desai, 2011; Mirman & Graziano, 2012; Schwartz et al., 2011). One, linked with the ATL, is thought to represent relationships between objects based on similarity in their S-M properties. A second system, supported by VPC, is thought to represent thematic or associative relations between items through sensitivity to spatiotemporal co-occurrence. Evidence for this view includes different semantic error patterns in patients with ATL versus VPC lesions (Schwartz et al., 2011) and activation of VPC during “combinatorial” semantic tasks that involve extraction of a global meaning from a series of words. These include comprehension of sentences (Friederici, Meyer, & von Cramon, 2000; Humphries, Binder, Medler, & Liebenthal, 2006; Vandenberghe et al., 2002) and determining the conjoint meaning of two-word phrases such as “loud car” (Graves, Binder, Desai, Conant, & Seidenberg, 2010; Price, Bonner, Peelle, & Grossman, 2015). However, while there is clear evidence for VPC involvement in sentence-level processing, this area frequently *deactivates* during single-word semantic processing (Humphreys et al., 2015; Humphreys & Lambon Ralph, 2014). This suggests that its function is distinct from that of ATL, which shows robust activation for single-word as well as sentence-level semantics. An alternative view holds that VPC acts as a short-term information buffer, maintaining aspects of recent experience that may be relevant to ongoing processing (Humphreys & Lambon Ralph, 2014; Jonides et al., 1998; Lerner, Honey, Silbert, & Hasson, 2011; Vilberg & Rugg, 2008; Wagner, Shannon, Kahn, & Buckner, 2005). On this view, VPC is important for semantic processing not because it is a long-term knowledge store but because it stores temporary information about recent context, which is important for comprehension beyond the level of single words.

Our model suggests a potential way to reconcile these different views. The implemented model is most consistent with the short-term buffer view of VPC function, in that the context layer acts a passive buffer that retains the previous state of the hub. This element of the model is critical for the context-dependent processing (e.g., the effects of cues in Simulation 1) but not for the processing of single words out of context (e.g., Simulations 2 and 3, where the activity on this layer is randomized prior to every trial).

However, one could envisage a more complex mode in which the context layer is not simply a passive store but instead plays a more direct role in mapping between words, S-M properties and predictions. Crucially, in order to maintain sensitivity to prior context, this layer would need to integrate inputs over a slower timescale than the ATL hub.^3^ In this hypothetical model, the context units would acquire representations of meaning, but they would be sensitive to spatiotemporal statistics over a longer timescale than those captured by the ATL hub. As a consequence, it is likely that they would play a disproportionate role in coding the semantics of temporally extended events, as envisaged by the idea of a hub for event knowledge (Binder & Desai, 2011; Mirman & Graziano, 2012; Schwartz et al., 2011). This potential account of VPC function is appealing for two other reasons. First, functional neuroimaging studies indicate that VPC does respond strongly to temporally extended streams of meaningful information (e.g., stories and movies) and, crucially, integrates information over a longer time scale than earlier sensory processing regions (Hasson, Yang, Vallines, Heeger, & Rubin, 2008; Lerner et al., 2011; Tylén et al., 2015). Second, if this role is assumed to extend beyond the semantic domain, then it provides a parsimonious explanation for VPC involvement in other types of processing, such as episodic memory for events and syntactic and arithmetical processing, all of which require sensitivity to the structure of temporally extended sequences (Humphreys & Lambon Ralph, 2014).

The final element of the model is the control processes that are necessary for the model to select from multiple response options in forced-choice tasks. Semantic control has been associated with a network of regions that include inferior frontal gyrus (IFG), posterior middle temporal gyrus, and the intraparietal sulcus, although most attention has been focused on the left IFG, which displays the most robust activation in functional neuroimaging studies (Badre et al., 2005; Bedny et al., 2008; Noonan et al., 2013; Rodd et al., 2005; Thompson-Schill et al., 1997; Whitney et al., 2011a, 2011b; Zempleni et al., 2007). Within the IFG, a division of labor has been proposed, whereby the most anterior portion (pars orbitalis, also known as Brodmann Area 47) is specialized for cognitive control during semantic processing while the posterior section (pars triangularis and opercularis or BA 44/45) has a domain-general role in response selection, which extends beyond semantics to other linguistic and nonlinguistic domains (Badre et al., 2005; Gold et al., 2006). This is supported by the structural connectivity of the region. BA47 has direct connections with the ATL hub region via the uncinate fasciculus while BA 44/45 demonstrates a broader pattern of connectivity with temporal and parietal regions (Binney, Parker, & Lambon Ralph, 2012). Badre and colleagues have proposed that these regions perform distinct roles in semantic control (Badre et al., 2005; Badre & Wagner, 2002, 2007). BA47 is thought to regulate activity in the semantic system through top-down controlled retrieval. In contrast, BA 44/45 is thought to be responsible for resolving competition between possible responses postretrieval. While the second process might govern behavior in a range of cognitive domains, the first appears to be more specific to semantic processing.

Our model makes specific predictions about the role of BA47 during semantic processing. If this region is responsible for controlled retrieval, we would expect it to be exhibit functional connectivity with the ATL during semantic tasks, reflecting its top-down influence on the activation of hub representations. Furthermore, this connectivity should be strongest when participants are required to activate nondominant or weak aspects of semantic knowledge. It is also important to note that we implemented semantic control as a two-stage process. We focused mainly on implementing a mechanism for controlled retrieval but we also included a stochastic response selection stage. To simulate SA, we disrupted both elements because the lesions that give rise to this condition typically encroach on both the anterior and posterior parts of IFG (Hoffman et al., 2010; Noonan et al., 2010). However, the model predicts that multiple, neuroanatomically distinct processes contribute to semantic control and this prediction could be tested by using TMS to disrupt the function of anterior versus posterior IFG. Finally, we note that some patients show impaired ability in controlled retrieval (i.e., poor ability to match weakly related concepts), despite having no damage to IFG (Noonan et al., 2010; Thompson, 2012). The deficit in these cases appears to arise from damage to the posterior components of the semantic control network. The function of these regions is poorly understood and is an important target for future investigations.

### Future Directions

In this final section, we discuss aspects of semantic processing that the model does not address at present and consider how these might be captured under our approach. One important aspect of language processing not currently addressed is the acquisition of syntax. Our model is presented with sequences of co-occurring nouns but has no exposure to other parts of speech, or indeed to event structure or other aspects of sentence processing. It is important to note this characteristic is shared with many of the existing models from the two modeling traditions that inspired the current project. Computational models of object semantics typically focus exclusively on the representations of individual object concepts (e.g., Chen et al., 2017; Devlin, Gonnerman, Andersen, & Seidenberg, 1998; Dilkina et al., 2008; Farah & McClelland, 1991; Plaut, 2002; Rogers et al., 2004; Schapiro et al., 2013; Tyler et al., 2000). Likewise, statistical models based on the distributional principle have often taken a “bag of words” approach that takes into account the propensity for words to occur in proximity to one another but disregards the order in which they occur (though some models have taken word order into account; Griffiths, Steyvers, Blei, & Tenenbaum, 2004; Jones & Mewhort, 2007). Taking our cue from these approaches, we restricted the model to processing noun sequences. This approach has been sufficient to provide a good fit to our target neuropsychological data, which concerned comprehension of individual words rather than sentences. Clearly, however, it is a gross oversimplification of language use in the real world. Many of the relationships between concepts are structured in terms of the roles they play in events, and these can be inferred from syntactic structure but not from mere co-occurrence. For example, mugs and glasses share many properties and this allows them to play similar roles in drink-making event sequences, to the extent that one can usually be substituted for the other. Mugs and coffee, on the other hand, frequently co-occur in the same context but they play different roles and, relatedly, have very different properties. Our model’s failure to take this information into account could result in “illusory feature migrations,” whereby properties of mugs are incorrectly generalized to coffee simply because they occur in the same contexts (Jones & Recchia, 2010). Similar constraints apply to the understanding of abstract words. For example, the words *journey* and *distance* have distinct meanings, despite frequently occurring in similar contexts, because they play different roles in the contexts in which they are used (one can measure the distance of a journey, but not the journey of a distance).

That said, there is no reason in principle why our model could not acquire representations that incorporate syntactic and role-based information, if trained with an appropriately structured corpus. Recurrent architectures of the kind we have used to represent context have been applied extensively to the study of sentence comprehension (Elman, 1990; St. John & McClelland, 1990). Such models readily acquire syntactic knowledge through sensitivity to statistical regularities in temporal structure. For example, a simple recurrent network presented with sentences will learn rapidly that verbs are typically followed by nouns and will represent these two classes as highly distinct from one another (Elman, 1990). We therefore see the present work as an important advance toward a model that extracts semantic information from full sentences while simultaneously binding this sequential statistical information with S-M experience.

A second simplification in the model concerns the representational basis of abstract words. We have adopted the most clearly articulated position in the literature: that knowledge of concrete and abstract words can inferred through their use in language, but only concrete words are directly associated with aspects of nonverbal S-M experience (Barsalou et al., 2008; Paivio, 1986). In addition, recent studies have indicated that the abstract-concrete continuum contains multiple underpinning distinctions and dimensions (Leshinskaya & Caramazza, 2016; Vigliocco et al., 2014). These include the fact that a set of abstract words are more strongly associated with emotional arousal than concrete words (Kousta et al., 2011; Vigliocco et al., 2014). Thus, it is likely that the representations of some abstract words are shaped not only by their linguistic use but by their association with particular emotional states, just as concrete words are associated with particular S-M experiences. Another subset of abstract words appears to be linked closely with representations of spatial and temporal magnitude (Troche, Crutch, & Reilly, 2014). These other potential influences on abstract word comprehension are not included in our model. However, the hub-and-spoke framework could potentially accommodate such influences by assuming that spoke regions that code emotional states and representations of magnitude also influence the development of conceptual representations in the hub (Binney, Hoffman, & Lambon Ralph, 2016; Rice et al., 2015). Indeed, the ATL hub region has direct structural connections with parts of the limbic system involved in emotion processing (Binney et al., 2012; Von Der Heide, Skipper, Klobusicky, & Olson, 2013). The effects of such additional sources of information on the organization of the semantic space is an interesting question that awaits investigation.

We also note that our model was not intended to test specific predictions about the timing of semantic processes. Although the cognitive neuroscience of semantic cognition has tended to focus on its spatial distribution throughout the brain, EEG and MEG studies provide complementary information on the timing of contributions from different regions. These suggest that ATL hub regions become activated as early as 200-ms postonset in lexical-semantic tasks (Chen, Davis, Pulvermüller, & Hauk, 2015; Hauk, 2016; Marinkovic et al., 2003), which is consistent with the central, intermediary role played by the hub in the model. Other studies suggest that the processing of words engages distributed linguistic information more rapidly than it does S-M representation (Barsalou et al., 2008). Although our model does not make specific predictions about timing, we believe that connectionist approaches more generally are well-suited to addressing these challenges, particularly those that have adopted neurally plausible activation dynamics (Blouw et al., 2015; Laszlo & Plaut, 2012).

Finally, we note that our treatment of semantic control has focused on one particular aspect, controlled retrieval. This is a critical ability because it allows individuals to identify connections between concepts which may initially appear unrelated. We tested this ability in a task which participants were asked to identify weak semantic relationships from various presented alternatives. But what value does such a process have in the real world, in which the alternatives are not so neatly presented? Our view is that when we encounter ambiguous stimuli, there are often multiple cues available, either in the environment or retrieved from our existing knowledge, that could potentially disambiguate the stimulus. Controlled retrieval is useful in finding the appropriate cue to aid our understanding. Imagine, for example, that you come across a friend in a supermarket while he is in the middle of a conversation with another acquaintance. You hear your friend say “I’m worried about its bark.” How do you make sense of this statement, without having heard the rest of the conversation? One possibility is that relevant constraining information is available among the items in your friend’s shopping basket. The presence of dog food could direct the semantic system toward one interpretation of *bark*, while the presence of weed killer would push the system toward a different interpretation. In other cases, the disambiguating information might be retrieved from memory. For example, if one of the salient facts you know about your friend is that they own a dog, this could serve as the additional information that drives the semantic system toward the relevant part of semantic space. In both of these examples, the disambiguating cues must be selected from a wide range of potentially relevant information. This, we believe, is the value of controlled retrieval in everyday life: for identifying which pieces of information cohere with one another, thus helping us to make sense of a complex world.

This mechanism may also be useful in the processing of metaphors and analogies. Although analogical reasoning was not a specific target of our model, a recent connectionist model has accounted for impairment in this domain in prefrontal and anterior temporal patients, using similar basic principles (Kollias & McClelland, 2013). In Kollias and McClelland’s (2013) model, completion of verbal analogy problems (e.g., Puppy is to dog as kitten is to what?) hinged on the ability of the network to process all of the elements of the problem simultaneously. Prefrontal damage in the model was simulated by preventing the network from considering all parts of the problem together, much as the removal of controlled retrieval in our model prevented the semantic hub from being influenced by all possible response options. More generally, comprehension of novel metaphors (e.g., the classroom was a zoo) requires people to identify which aspect of meaning from the metaphor’s source can be cogently applied to the target. This constrained search for a shared aspect of meaning is precisely the function of the controlled retrieval mechanism. Thus, while a detailed consideration of metaphor is beyond the scope of the current model, the approach to semantic control we have outlined may have some utility in this domain.

Although controlled retrieval is an important tool in many situations, we also believe that other control processes make important contributions to semantic cognition. Many tasks require inhibition of prepotent associations to direct attention to specific aspects of meaning. As discussed earlier, these goal-driven biases may be achieved through the influence of representations of task set on activity in the semantic system (e.g., Dilkina et al., 2008; Plaut, 2002). We have also not attempted to specify in detail the processes involved in response selection, which is a particular source of difficulty for patients with SA. The degree to which these different elements of semantic control rely on unitary versus diverse neural substrates is unclear at the present time. Only by investigating the underlying mechanisms will we be able to develop a unified theory of semantic cognition that addresses not only the representation of semantic knowledge but also its appropriate use.

## Figures and Tables

**Table 1 tbl1:** Analyses of Human and Model Performance in Simulation 1

Effect	Human			Model		
	*df*	*F*	*p*	*df*	*F*	*p*
Impairment	1, 13	74.3	<.001	1, 9	2692	<.001
Dominance	1, 13	11.58	<.005	1, 9	218	<.001
Cue	2, 26	35.88	<.001	2, 18	983	<.001
Dominance × Impairment	1, 13	6.16	<.005	1, 9	69.8	<.001
Cue × Impairment	2, 26	29.51	<.001	2, 18	226	<.001
Dominance × Cue × Impairment	2, 26	8.0	<.005	2, 18	17.1	<.001
*Note.* Analyses of human data were reported by Noonan et al. (2010). Analyses of model data treated each of the ten trained models (each trained in the same way but initialized with different random weights) as a separate case. Impairment was treated as within-models factor, since each model was tested before and after damage.						

**Table 2 tbl2:** Regression Analysis of Probe-Target Co-Occurrence Rates in Simulation 2

Effect	*R*^*2*^	*p*	β	*p*
Neuropsychological test	.12	.010		
Frequency			.33	.014
Imageability			.08	.467
Semantic diversity			−.35	.011
Model test	.15	.038		
Frequency			.31	.078
Imageability			.10	.456
Semantic diversity			−.46	.012

**Table 3 tbl3:** Correlation Matrices for Human and Model Data in Simulation 2

Effect	Imageability	Semantic diversity	*SD* accuracy	SA accuracy
Human data				
Frequency	−.021	.596**	.528**	−.053
Imageability	—	−.361**	.413**	.465**
Semantic diversity		—	.069	−.438**
*SD* accuracy			—	.412**
Model data				
Frequency	−.086	.465**	.616**	.061
Imageability	—	−.327*	.207	.352*
Semantic diversity		—	.246	−.352*
*SD* accuracy			—	.270*
*Note.* Human data were originally reported by Hoffman, Rogers, and Ralph, (2011b).				
* *p* < .05. ** *p* < .001.				

**Table 4 tbl4:** Regression Analyses of Human and Model Data in Simulation 2

Effect	*R*^*2*^	*p*	β	*p*
Human—SD	.478	<.001		
Frequency			.65	<.001
Imageability			.36	<.001
Semantic diversity			−.19	.081
Human—SA	.333	<.001		
Frequency			.23	.042
Imageability			.30	.002
Semantic diversity			−.47	<.001
Model—SD	.449	<.001		
Frequency			.62	<.001
Imageability			.28	.017
Semantic diversity			.05	.700
Model—SA	.242	.003		
Frequency			.27	.062
Imageability			.25	.066
Semantic diversity			−.40	.010
*Note.* SD = semantic dementia; SA = semantic aphasics.				

## Footnotes

1 This choice had no major effect on the results reported in this article: Similar results were obtained in alternative model in which homonyms were abstract.

2 In addition, as we describe in Simulation 2, *all* concepts were associated with some contextual variability, as they all occurred in at least three different topics (*M* = 7.6; the most variable word, *problem*, occurred in 23 topics).

3 We are grateful to David Plaut for this suggestion.
